# The highly selective oxidation of cyclohexane to cyclohexanone and cyclohexanol over VAlPO_4_ berlinite by oxygen under atmospheric pressure

**DOI:** 10.1186/s13065-018-0405-6

**Published:** 2018-04-04

**Authors:** Yun Hong, Dalei Sun, Yanxiong Fang

**Affiliations:** Department of Chemical Engineering and Light Industry, Guangdong University of Technology, Guangzhou, 510006 China

**Keywords:** Oxidation, Cyclohexane, Heterogeneous catalyst, Berlinite

## Abstract

**Background:**

The oxidation of cyclohexane under mild conditions occupies an important position in the chemical industry. A few soluble transition metals were widely used as homogeneous catalysts in the industrial oxidation of cyclohexane. Because heterogeneous catalysts are more manageable than homogeneous catalysts as regards separation and recycling, in our study, we hydrothermally synthesized and used pure berlinite (AlPO_4_) and vanadium-incorporated berlinite (VAlPO_4_) as heterogeneous catalysts in the selective oxidation of cyclohexane with molecular oxygen under atmospheric pressure. The catalysts were characterized by means of by XRD, FT-IR, XPS and SEM. Various influencing factors, such as the kind of solvents, reaction temperature, and reaction time were investigated systematically.

**Results:**

The XRD characterization identified a berlinite structure associated with both the AlPO_4_ and VAlPO_4_ catalysts. The FT-IR result confirmed the incorporation of vanadium into the berlinite framework for VAlPO_4_. The XPS measurement revealed that the oxygen ions in the VAlPO_4_ structure possessed a higher binding energy than those in V_2_O_5_, and as a result, the lattice oxygen was existed on the surface of the VAlPO_4_ catalyst. It was found that VAlPO_4_ catalyzed the selective oxidation of cyclohexane with molecular oxygen under atmospheric pressure, while no activity was detected on using AlPO_4_. Under optimum reaction conditions (i.e. a 100 mL cyclohexane, 0.1 MPa O_2_, 353 K, 4 h, 5 mg VAlPO_4_ and 20 mL acetic acid solvent), a selectivity of KA oil **(**both cyclohexanol and cyclohexanone) up to 97.2% (with almost 6.8% conversion of cyclohexane) was obtained. Based on these results, a possible mechanism for the selective oxidation of cyclohexane over VAlPO_4_ was also proposed.

**Conclusions:**

As a heterogeneous catalyst VAlPO_4_ berlinite is both high active and strong stable for the selective oxidation of cyclohexane with molecular oxygen. We propose that KA oil is formed via a catalytic cycle, which involves activation of the cyclohexane by a key active intermediate species, formed from the nucleophilic addition of the lattice oxygen ion with the carbon in cyclohexane, as well as an oxygen vacancy formed at the VAlPO_4_ catalyst surface.
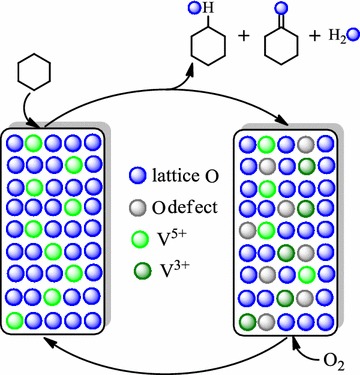

**Electronic supplementary material:**

The online version of this article (10.1186/s13065-018-0405-6) contains supplementary material, which is available to authorized users.

## Introduction

With the development of petrochemical industry, the oxidation of cyclohexane under mild conditions, with molecular oxygen or air, is of great interest [1, 2]. In the autoxidation of cyclohexane, most industrial processes are involved with the usage of soluble transition metal catalysts, including vanadium oxide, at 423 ~ 453 K and afford the mixture of cyclohexanol, cyclohexanone and dicarboxylic acids, which is formed by further oxidation of cyclohexanone and cyclohexanol [2, 3]. However, the use of soluble metal catalysts in these systems often requires a tedious catalyst separation step [4]. Thus, it is necessary to develop effective recyclable heterogeneous catalysts for selective oxidation of cyclohexane by O_2_.

The AlPO-n families are divided into two groups: dense-phase berlinite or tridymite and porous aluminophosphate molecular sieve [5]. Berlinite is the nonporous and stable phase of polymorphous aluminophosphates [6] and potentially mainly used in functional material fields, such as acoustic wave device, memory glass [7] and piezoelectric material [8], as well as, high-performance sealants for corrosion- and wear-resistant coatings [9]. Porous aluminophosphates and their derivates (MeAPO-n) incorporated with transition metals were widely used as catalysts, including VAPO-5 molecular sieves [3]. For example, they have been frequently used as catalysts for the selective oxidation of cyclohexane to produce cyclohexanol and cyclohexanone [10, 11]. At the same time, the heterogeneous MeAPO-n molecular sieve as catalysts is a very controversial issue and it is generally recognized that metals are leached into the polar solvents, such as acetic acid [12].

Berlinite is more stable than MeAPO-n molecular sieve [5, 6]. But they had seldom been applied in catalytic cyclohexane oxidation. Accordingly, we report the application for the first time as well as the preparation, characterization and catalytic performance in cyclohexane oxidation of a new VAlPO_4_ berlinite, in which vanadium was incorporated. It is found to be an active recyclable heterogeneous catalyst for the selective oxidation of cyclohexane with molecular oxygen under mild conditions.

## Experiment

### Catalyst preparation

Al(CH_3_COO)_3_·2H_2_O, H_3_PO_4_ (85% sol in water), and V_2_O_5_ were used as the sources of aluminum, phosphorus, vanadium, and triethyl amine (Et_3_N) was used as template. VAlPO_4_ berlinite was synthesized from the gel according to the following molar ratio: 0.02 V:0.92 Al:1.0 P:0.81 Et_3_N:30 H_2_O. During typical synthesis, Al(OAc)_3_ was hydrolyzed firstly at room temperature for 2 h, and aqueous solution of V_2_O_5_ and H_3_PO_4_ was added into the obtained solution. The formed mixture was stirred at room temperature for 2 h and Et_3_N were then added into the homogeneous gel at 273 K under vigorous stirring. Finally, the mixture was stirred at 273 K for another 3 h. The final gel was charged in a Teflon-lined autoclave and allowed to crystallize at 453 K for 48 h. The VAlPO_4_ berlinite was filtered and washed several times with deionized water until the pH value was 7. The crystals were dried at 373 K for 6 h and then calcined at 823 K for 10 h to remove the Et_3_N template.

VAlPO-5 molecular sieve was also synthesized according the method reported by Concepción et al. [3].

### Characterization

XRD was performed on a Brucker D8 Advance diffractometer with Cu K*α*1 radiation according to the scanning range of 2θ = 6–80° at a rate of 1°/min. Fourier transform infrared (FT-IR) spectroscopy was conducted on a Varian 3100 spectrometer in transmission mode with the resolution of 4 cm^−1^. The VAlPO_4_ specimen was mixed with KBr according to the weight ratio of 1:200 and pressed into pellets for measurement. The spectra were recorded as the accumulated results of 125 scans and the spectra of dry KBr were selected for background subtraction. X-ray photoelectron spectroscopy (XPS) was carried out on a Phi Quantum 2000 Scanning ESCA Microprobe with Al Kα radiation. A C1s binding energy of 284.6 eV was used as the reference. Microphotography and EDAX analyses were performed on a Philips SEM 505 instrument equipped with an EDAX detecting unit. Chemical analyses of V content were performed by atomic absorption spectroscopy (AAS) with a Varian AA240 spectrometer. The chemical compositions determined with EDAX were compared with the results obtained by XPS and the content of vanadium obtained by AAS analyses of the solutions prepared by thermal acid digestion of the sample.

### Catalytic reaction

The catalytic performance of VAlPO_4_ berlinite was tested through cyclohexane (≥ 99.5%, without further purification, Beijing Chem. Corp.) oxidation as model reaction with molecular oxygen under atmospheric pressure. The reaction was carried out at 348 K in a 250 mL three-neck flask equipped with a condenser. Typically, 80 g cyclohexane, 40 g acetic acid (used as solvent), 0.5 g cyclohexanone (used as initiator) and 0.5 g catalyst were added into the three-neck flask at room temperature. Then, the reactor was heated to the reaction temperature and the reaction solution was stirred with an external magnetic stirrer. At the reaction temperature, the reactor was charged with a flow of O_2_. The flow rate of the O_2_ was controlled in the way that bubbles of oxygen appeared in the solution and that no oxygen could be detected in the outlet of the condenser to ensure that oxygen was totally consumed by the oxidation of cyclohexane. After 6 h, the reaction stopped. After cooling down to room temperature, the reaction mixture was diluted with 20 g ethanol to produce a homogeneous solution and then the catalyst was separated through filtration. The filtration solution was used for composition analysis.

To examine the stability of the catalyst, the solution of product mixtures obtained from the oxidation of cyclohexane as mentioned above was filtered to remove the catalyst. The obtained solution was used directly as the reactant without the addition of catalyst, cyclohexanone and acetic acid and subjected to the oxidative reaction in the same condition: reaction temperature of 348 K, the oxidant of molecular oxygen and atmospheric pressure. After 10 h, the reaction stopped. The product mixture was sampled and analyzed.

The reaction products were analyzed by GC–MS and HPLC for identification (Additional files 1 and 2) . The quantitative analyses of cyclohexanol and cyclohexanone were carried out by Agilent 4890D gas chromatography with OV-1701 column (30 m × 0.25 mm × 0.3 µm) and the internal standard of methylbenzene. The carboxylic acids were analyzed on Agilent 1100 Series HPLC instrument with a 250 × 4.6 mm Microsorb-MV (C18) column and an ultraviolet detector. The analysis conditions were provided as follows: flow phase of water/methanol (10 ~ 30%)/KH_3_PO_4_ (5 mM), pH value (3 ~ 4) of flow phase adjusted with H_3_PO_4_ (25%), flow rate of 1.0 mL min^−1^, column temperature of 298 K and ultraviolet wavelength of 212 nm. The contents of by-products acid were determined according to external standard method and calculated according to the equation W_*sp*_= W_*st*_·A_*sp*_/A_*st*_× 100%, where *sp* and *st* indicated specimen and standard, respectively. The conversion rate of cyclohexane and the yield of cyclohexanol and cyclohexanone were calculated according to the converted cyclohexane.

The solid catalyst was separated by filtration and washed with 20 mL of acetone, and then dried at 373 K for 2 h after each reaction.

## Results and discussion

### Characterization

**Fig. 1:**
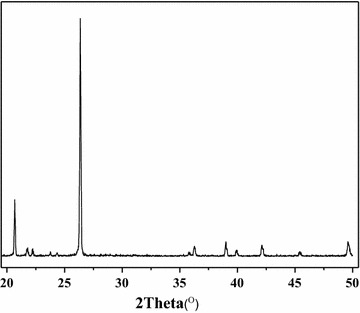
XRD pattern of the VAlPO_4_ catalyst

Figure 1 shows the XRD pattern of the VAlPO_4_ berlinite, which is totally consistent with that of standard berlinite (JCPDS No. 76-227). Other crystalline or amorphous phases were not detected.

**Fig. 2:**
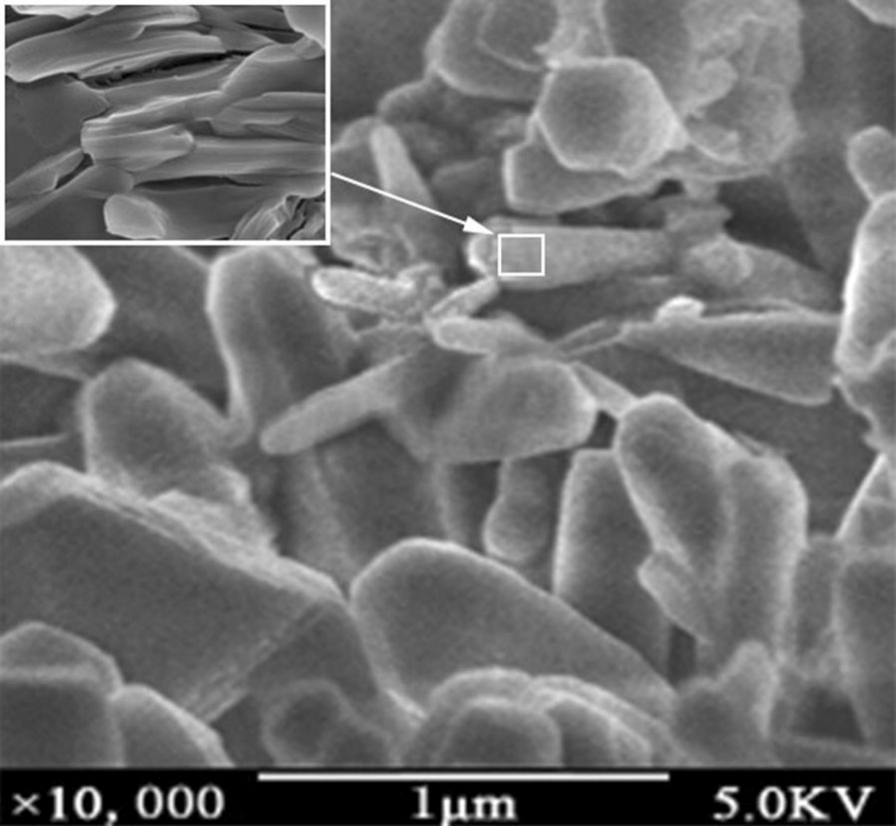
SEM pictures of the VAlPO_4_ catalyst

The microphotographs (Fig. 2) show the snowflake structure shape of VAlPO_4_ berlinite, without the presences of any other amorphous phases. The catalyst compositions determined by EDAX and AAS analyses are summarized as follows: 0.23 V_2_O_5_: 1.00 Al_2_O_3_: 1.14 P_2_O_5_ for VAlPO_4_ berlinite. The chemical composition determined by EDAX is in good agreement with those obtained by AAS analysis, indicating the uniform distribution of the vanadium in the VAlPO_4_ berlinite. The mapping of a 20 μm crystal of the sample at fifteen different points showed a practically constant composition, indicating the homogeneous distribution of vanadium in the crystal.

**Fig. 3:**
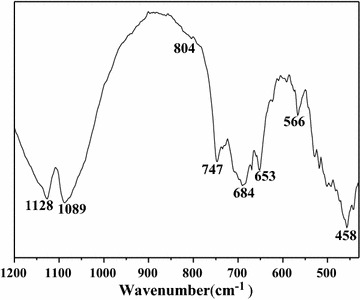
FT-IR spectrum of the VAlPO_4_ catalyst

After calcination at 823 K in air, according to the subsequent determination results by FT-IR spectroscopy (Fig. 3), the template was completely removed. The spectrum of the VAlPO_4_ catalyst exhibited the characteristic vibration absorptions of a berlinite structure [5, 6, 13–16], i.e. the bands at 1128 cm^−1^ are ascribed to the asymmetric Al-O and/or P-O stretching modes and the bands at 804 cm^−1^ are ascribed to the symmetric Al-O and/or P-O stretch in TO_4_ (T = Al or P) [5, 6, 15], the bands at 684 and 458 cm^−1^ are assigned to the Al-O and/or P-O bending modes [5, 15, 16], and some of which were shifted towards lower wavenumbers probably due to the incorporation of V into the berlinite framework. In addition, a few additional bands at 1089, 747, 684, 653, and 566 cm^−1^ were also detected in the VAlPO_4_ spectrum compared to that for AlPO_4_ [16–18]. Thus, the bands at 1089, 747, 684, 653, and 566 cm^−1^ should be caused by the incorporation of V into the berlinite and assigned to the vibrations of V-O-P [13, 19], providing further evidence for the incorporation of V into the berlinite framework.

**Fig. 4:**
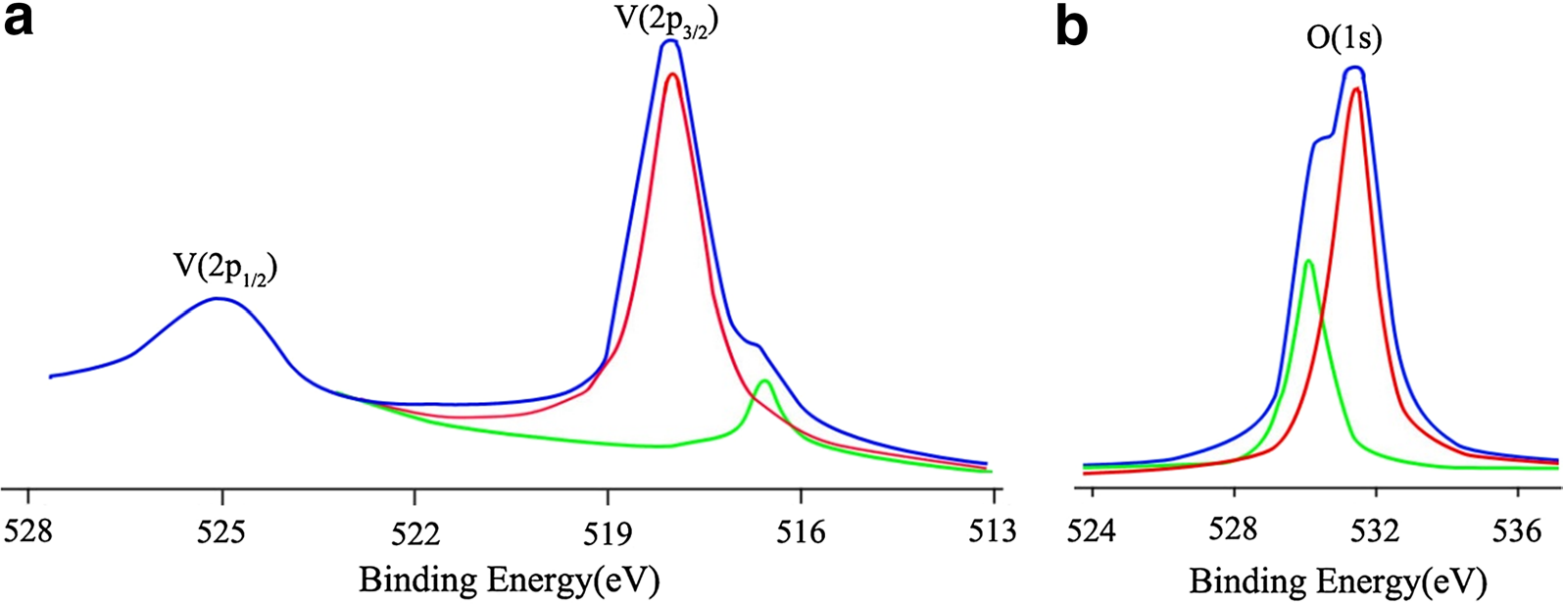
V2p (**a**) and O1s (**b**) XPS spectra of the VAlPO_4_ catalyst

The XPS measurement shows that the surface atomic composition of the VAlPO_4_ catalyst is V:Al:P:O = 1.0:4.4:5.0:20.0. The V2p and O1s XPS spectra are shown in Fig. 4a, b. The binding energy of the V2p_1/2_ and V2p_3/2_ peaks (Fig. 4a) is, respectively 524.7 and 517.6 eV in the VAlPO_4_ catalyst. Compared with the V2p1/2 and V2p3/2 signal for V_2_O_5_, that is respectively 525.8 eV and 518.3 eV [20, 21], those of the VAlPO_4_ catalyst slightly shifted toward lower binding energy, indicating that V(V) ions, replacing the Al(III) and/or P(V), are incorporated into the berlinite framework, resulting in oxygen vacancies in close vicinity to V(V), and possessed a higher tendency to draw electrons as compared to those in V_2_O_5_. Meanwhile, the O_1s_ signal for the VAlPO_4_ catalyst (Fig. 4b) is 532.2 eV, higher than that for V_2_O_5_ (BE = 531.6 eV) [20, 21]. The results further suggested that the lattice oxygen was existed on the surface of the VAlPO_4_ catalyst. Thus, the catalytic activity of vanadium oxide in oxidation reactions is improved.

### Cyclohexane oxidation

VAlPO_4_ berlinite catalyzed the oxidation of cyclohexane and the results were shown in Table 1. Leaching ratio of the metal into solution was checked by AAS analyses of the supernatant solution (see Table 1). It is found that no vanadium is leached into the solution. At the same time, the leaching tests showed that the reaction (Table 1) nearly stopped after the removal of the solid catalysts. For example, the reaction with neat cyclohexane and the supernatant after the removal of solid VAlPO_4_ berlinite showed the small additional conversion ratio (only 0.04%) during the 10 h leaching testing. The catalyst was recycled for three times without activity loss. At the same time, according to the method proposed by Concepción et al. [3], we prepared VAPO_4_ -5 molecular sieve and compared it with VAlPO_4_ berlinite as catalyst for the selective oxidation of cyclohexane with molecular oxygen under mild conditions. High metal leaching ratio was observed, which was consistent with previous results reported by Lin et al. [3, 4, 10–12]. In contrast, berlinite is more stable than porous aluminophosphate molecular sieve. Thus, The VAlPO_4_ berlinite is proved to be more stable than VAPO_4_-5 molecular sieve as heterogeneous catalyst for the selective oxidation of cyclohexane with molecular oxygen under atmospheric pressure.

**Table 1:** Catalytic oxidation of cyclohexane over VAlPO_4_ berlinite and VAPO-5 molecular sieve

Catalyst	χ (%)^a^	Si (%)^c^			[V, Co and/or Mn] (ppb)^d^			χ (%)^b^
		Ol	One	Others	V	Co	Mn	
AlPO_4_	0	0	0	0	0	0	0	0
VAlPO_4_	5.9	69.2	28.6	1.8	11	–	–	0.04
VAPO-5	6.3	60.5	35.0	1.0	390	–	–	0.8
V_2_O_5_	2.1	51.3	44.6	4.1	610	–	–	1.1
VAlPO_4_^e^	5.7	68.7	28.9	2.4	15	–	–	0.09
CoAPO_4_ [22]	3.8	91.3	7.4	1.3	–	24	–	0.03
MnAPO_4_ [22]	4.1	93.6	5.6	0.8	–	–	0	0.01
CoMnAPO_4_ [22]	5.2	60.7	33.7	0.5	–	15	8	0.04

Cyclohexane 100 mL, VAlPO_4_ berlinite catalyst 5 mg, acetic acid solvent 40 mL, O_2_ pressure 0.1 MPa, 348 K, 4 h

^a,b^ χ: Cyclohexane conversion in normal and leaching test, respectively; ^c^ Si: Ol, cyclohexanol; One, cyclohexanone; Others, C_4_–C_6_ diacids and their esters; ^d^ Concentrations of metal ion leaked into solution; ^e^ VAlPO_4_ berlinite catalyst recycled for the fifth time as a catalyst in the reaction batch

For comparison, under the same reaction conditions for the oxidation of cyclohexane, we studied the catalyst of AlPO_4_ berlinite without the incorporation of V and the catalyst of VAlPO_4_ berlinite. AlPO_4_ berlinite did not exhibit any significant activity. The higher activity of VAlPO_4_ berlinite may be attributed to that V(V) ions are incorporated into the berlinite framework, resulting in oxygen vacancies in close vicinity to V(V), and possessed a higher tendency to draw electrons as compared to those in V_2_O_5_. In order to check the reusability of the catalyst, it was recycled for five times without activity loss. Thus, in the oxidation of cyclohexane with molecular oxygen under mild conditions, compared with other berlinite catalysts, such as AlPO_4_, CoAlPO_4_ and MnAlPO_4_, VAlPO_4_ berlinite showed higher catalytic activity. Then, Factors influencing the reaction using VAlPO_4_ berlinite as catalyst were studied systematically, with a possible reaction mechanism also proposed.

#### Effect of solvents

Table 2 presents the results of oxidation of cyclohexane with molecular oxygen in the absence and presence of various solvents (acetic acid, *N*-propylsulfonic acid pyridinium tetrafluoroboborate (IL), or acetonitrile), using VAlPO_4_ as catalyst, a reaction time of 3 h and a reaction temperature of 353 K. All the batches consisted of 100 mL cyclohexane, 0.1 MPa O_2_, 5 mg VAlPO_4_ and 20 mL solvent. It was found that in the absence of solvent, the conversion of cyclohexane, the selectivity to KA oil were only 3.0 and 94.3%, respectively. When a solvent was employed, the conversion of cyclohexane, the selectivity to KA oil (both cyclohexanol and cyclohexanone) increased to above 4.1 and 95.8%, respectively. This indicates that the solvent stimulated the oxidation of cyclohexane with molecular oxygen. The stimulation by the solvent was in the order acetic acid >*ψ* IL >*ψ* acetonitrile >*ψ* no solvent. The above result reveals that acetic acid as solvent is favorable for the oxidation of cyclohexane with molecular oxygen, which is probably due to the cyclohexane has better solubility in acetic acid [23].

**Table 2:** Conversions of cyclohexane and selectivities to products in different solvents

Solvent	Conversion (%)	Selectivity (%)			
		KA oil^a^	Acids^b^	Esters^c^	CHHP^d^
Without	3.0	94.3	0.3	0.2	5.2
Acetic acid	6.8	97.2	1.6	0.5	0.7
IL	5.9	96.3	1.2	0.9	1.6
Acetonitrile	4.1	95.8	1.2	1.3	1.7

Cyclohexane 100 mL, VAlPO_4_ berlinite catalyst 5 mg, acetic acid solvent 40 mL, O_2_ pressure 0.1 MPa, 353 K, 4 h

^a^Cyclohexanol and cyclohexanone; ^b^ C_4_–C_6_ diacids; ^c^ synthesized by the reaction of C_4_–C_6_ diacids and cyclohexanol; ^d^ cyclohexyl hydroperoxide

#### Effect of reaction temperature

**Fig. 5:**
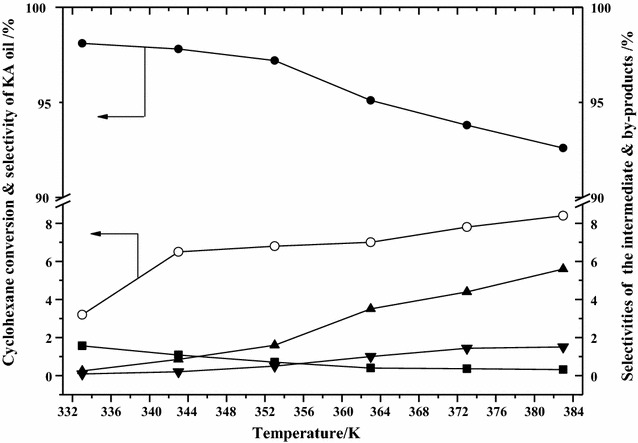
Effect of reaction temperature on cyclohexane conversion, selectivities for the main product, intermediate product, and by-products. Reaction conditions: cyclohexane 100 mL, VAlPO_4_ berlinite catalyst 5 mg, acetic acid solvent 40 mL, O_2_ pressure 0.1 MPa, reaction time: 4 h. (White circle) cyclohexane conversion; (Black circle), (Black square), (Black up-pointing triangle) and (Black down-pointing triangle) selectivity for KA oil, CHHP, acids and esters, respectively. KA oil: cyclohexanol and cyclohexanone; CHHP: cyclohexyl hydroperoxide; acids: C_4_–C_6_ diacids; esters: synthesized by the reaction of C_4_–C_6_ diacids and cyclohexanol

Figure 5 presents the effect of reaction temperature on cyclohexane conversion and selectivities for the main product, the intermediate product, and by-products. On increasing reaction temperature, the conversion of cyclohexane increased rapidly over the temperature range 333–373 K, and only slightly at temperatures higher than 373 K, approaching its maximum of 8.2%. The above results indicate that the elevation of reaction temperature promoted the conversion of cyclohexane. The selectivity of KA oil increased with on moving from 333 to 353 K, attaining a maximum of 97.2% at 353 K, before decreasing at higher temperatures. The selectivity for the intermediate product cyclohexyl hydroperoxide (CHHP) first increased and then decreased during the reaction temperature range 333–383 K. This could be due to the fact that a higher temperature accelerates the decomposition of the intermediate CHHP to main product KA oil [24]. The selectivities for by-products both acids and esters increased with the increase of reaction temperature. For all the reaction temperature points tested, the selectivity for main product KA oil was much larger than that for both the intermediate CHHP and by-products (acids and esters). Although a higher conversion of cyclohexane could be attained at high temperature, too high a temperature reduced the selectivity of KA oil—possibly due to the further oxidation of KA oil into acid and the synthesis of ester by the reaction from both acid and cyclohexanol [24]. Thus, the optimum reaction temperature for the oxidation of cyclohexane with molecular oxygen using under atmospheric pressure is around 353 K.

#### Effect of reaction time

**Fig. 6:**
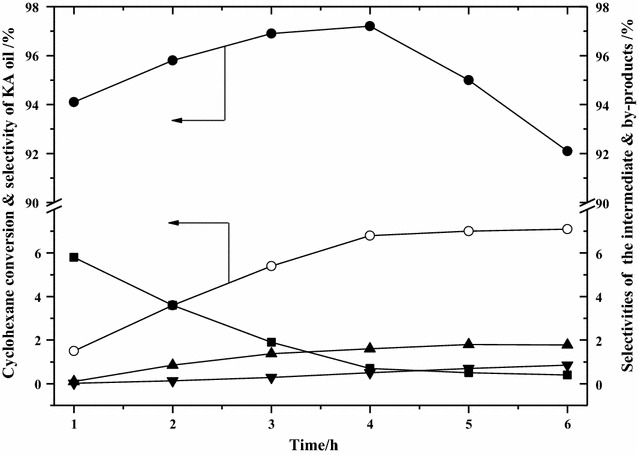
Effect of reaction time on cyclohexane conversion, selectivities for the main product, intermediate product, and by-products. Reaction conditions: cyclohexane 100 mL, VAlPO_4_ berlinite catalyst 5 mg, acetic acid solvent 40 mL, O_2_ pressure 0.1 MPa, reaction time: 4 h. (White circle) cyclohexane conversion; (Black circle), (Black square), (Black up-pointing triangle) and (Black down-pointing triangle) selectivity for KA oil, CHHP, acids and esters, respectively. KA oil: cyclohexanol and cyclohexanone; CHHP: cyclohexyl hydroperoxide; acids: C_4_–C_6_ diacids; esters: synthesized by the reaction of C_4_–C_6_ diacids and cyclohexanol

Figure 6 outlines the effect of reaction time on cyclohexane conversion and selectivities for the main product, the intermediate product, and by-products. With increasing reaction time, the cyclohexane conversion increased quickly within 4 h and only slightly over longer reaction times, reaching a value of nearly 7%. The selectivity of KA oil increased, followed by a decrease, with a maximum value of 97% being achieved at a reaction time of 4 h. On prolonging the reaction timeframe, the selectivity for the by-products both acids and esters increased gradually, while that for the intermediate product CHHP decreased slowly. These results indicate that a longer reaction time promoted the decomposition of the intermediate CHHP to the main product KA oil, but a too long reaction time resulted in the further oxidation of KA oil into acid and the synthesis of ester by the reaction from both acid and cyclohexanol. Thus, the optimum reaction time is suggested as being 4 h.

### Mechanistic consideration to the oxidation of cyclohexane with molecular oxygen over the VAlPO_4_ catalyst

**Scheme 1:**
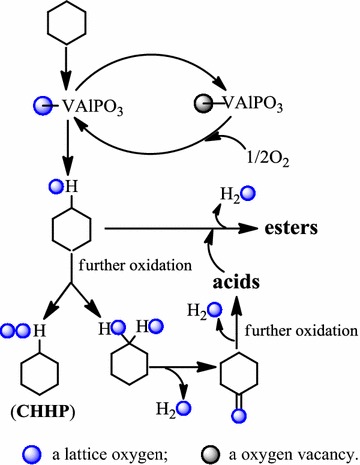
Possible mechanism for the formation of KA oil, CHHP, acids and esters via the oxidation of cyclohexane with molecular oxygen using VAlPO_4_ as a catalyst. KA oil: cyclohexanol and cyclohexanone; CHHP: cyclohexyl hydroperoxide; acids: C_4_–C_6_ diacids; esters: synthesized by the reaction of C_4_–C_6_ diacids and cyclohexanol

Although mechanistic studies on the oxidation of cyclohexane with molecular oxygen in the presence of a VAlPO_4_ catalyst are still in progress, it can be surmised that the reaction pathway may involve a catalytic cycle that involves a number of steps (Scheme 1). At first, the carbon in cychohexane is attacked by the nucleophilic lattice oxygen ion of VAlPO_4_ catalyst, forming a reaction product cyclohexanol. Meanwhile, the V in VAlPO_4_ catalyst lattice is reduced, leaving an oxygen vacancy at the VAlPO_4_ catalyst surface. Such an oxygen vacancy is then filled with oxygen from the gas phase, which simultaneously reoxidizes the reduced V of VAlPO_4_ catalyst lattice results in the recovery of the VAlPO_4_ catalyst. Similarly, both cyclohexanone product and cyclohexyl hydroperoxide (CHHP) intermediate could be resulted from further oxidation cyclohexanol by molecular oxygen in the presence of a VAlPO_4_ catalyst [24, 25]. Then, additional further oxidation of cyclohexanone would end up in ring-opened acid by-products,which can be esterified by cyclohexanol, generating the ester by-products [24, 25]. It must be noted that the oxidation depth of cyclohexane is closely related to the reaction conditions, especially the reaction temperature. In general, the depth of cyclohexane oxidation increases with the increase of the reaction temperature. For this reason, only a lower than 1% acids by-products was formed because of cyclohexane oxide deeply during the manufacture of KA oil (cyclohexanol and cyclohexanone) by the oxidation of cyclohexane over the VAlPO_4_ catalyst under mild conditions (i.e. 333 ~ 383 K, atmospheric pressure).

## Conclusions

A new material, VAlPO_4_ berlinite, has been prepared and characterized. It is proved that the vanadium is incorporated into the framework of AlPO_4_ berlinite. The catalytic activity of VAlPO_4_ berlinite in cyclohexane oxidation is higher than that of CoAPO_4_ or MnAPO_4_ under the same conditions and similar loads of cobalt and manganese. Furthermore, AlPO_4_ berlinite without the incorporation of any metal is not active in the oxidation of cyclohexane with molecular oxygen under mild conditions. Although the catalytic activity of VAPO_4_-5 molecular sieve is similar to that of VAlPO_4_ berlinite under the same conditions, high leaching ratio of vanadium into the solution is observed when VAPO_4_-5 molecular sieve is used as catalyst. Meanwhile, the mechanism for the oxidation of cyclohexane with molecular oxygen over the VAlPO_4_ catalyst may have resulted from a catalytic cycle involving a key active intermediate species-formed from the nucleophilic addition of the lattice oxygen ion with the carbon in cyclohexane—that leaves an oxygen vacancy at the VAlPO_4_ catalyst surface, which further splits oxygen molecules into atoms and then acts as a reservoir that can take up these atoms and then release them to form molecules. In conclusion, VAlPO_4_ berlinite is an efficient recyclable heterogeneous catalyst for the selective oxidation of cyclohexane with molecular oxygen under mild conditions.

## Additional files

**Additional file 1.** The GC-MS of reaction products.

**Additional file 2.** The HPLC of reaction products.
