# Supplementary material for: The highly selective oxidation of cyclohexane to cyclohexanone and cyclohexanol over VAlPO4 berlinite by oxygen under atmospheric pressure
Source: Chem Cent J. 2018 Apr 4;12:36. doi: 10.1186/s13065-018-0405-6 (PMC5884748; doi:10.1186/s13065-018-0405-6)
Supplement: Supplementary file 1 — Additional file 1. The GC-MS of reaction products. [file 13065_2018_405_MOESM1_ESM.pdf]

谱库检索报告

数据路径　：D:\msdchem\1\data\徐雄发\方岩雄-肖业鹏\
数据文件　：8.D
采集　　　：29 Apr 2014　16:46
操作者　　：
样品　　　：8
其他　　　：
ALS 样品瓶：8　样品乘积因子：1

检索库：　D:\DATABASE\DEMO.L　　　　　最小匹配度：　0
　　　　　C:\DATABASE\NIST11.L　　　　最小匹配度：　0

未知谱图：　顶点
积分事件：化学工作站积分器 - events.e

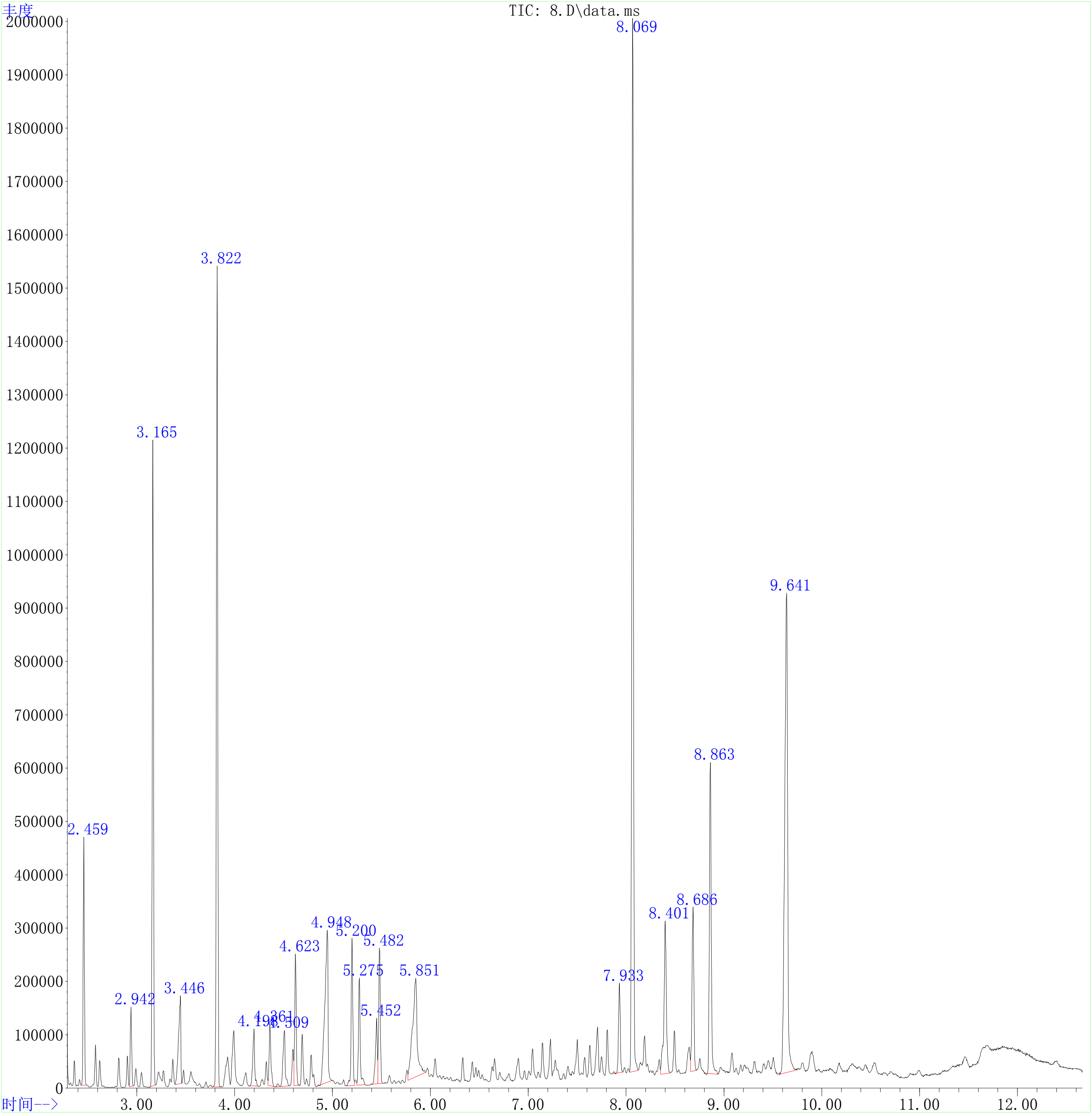

未知谱图基于顶点

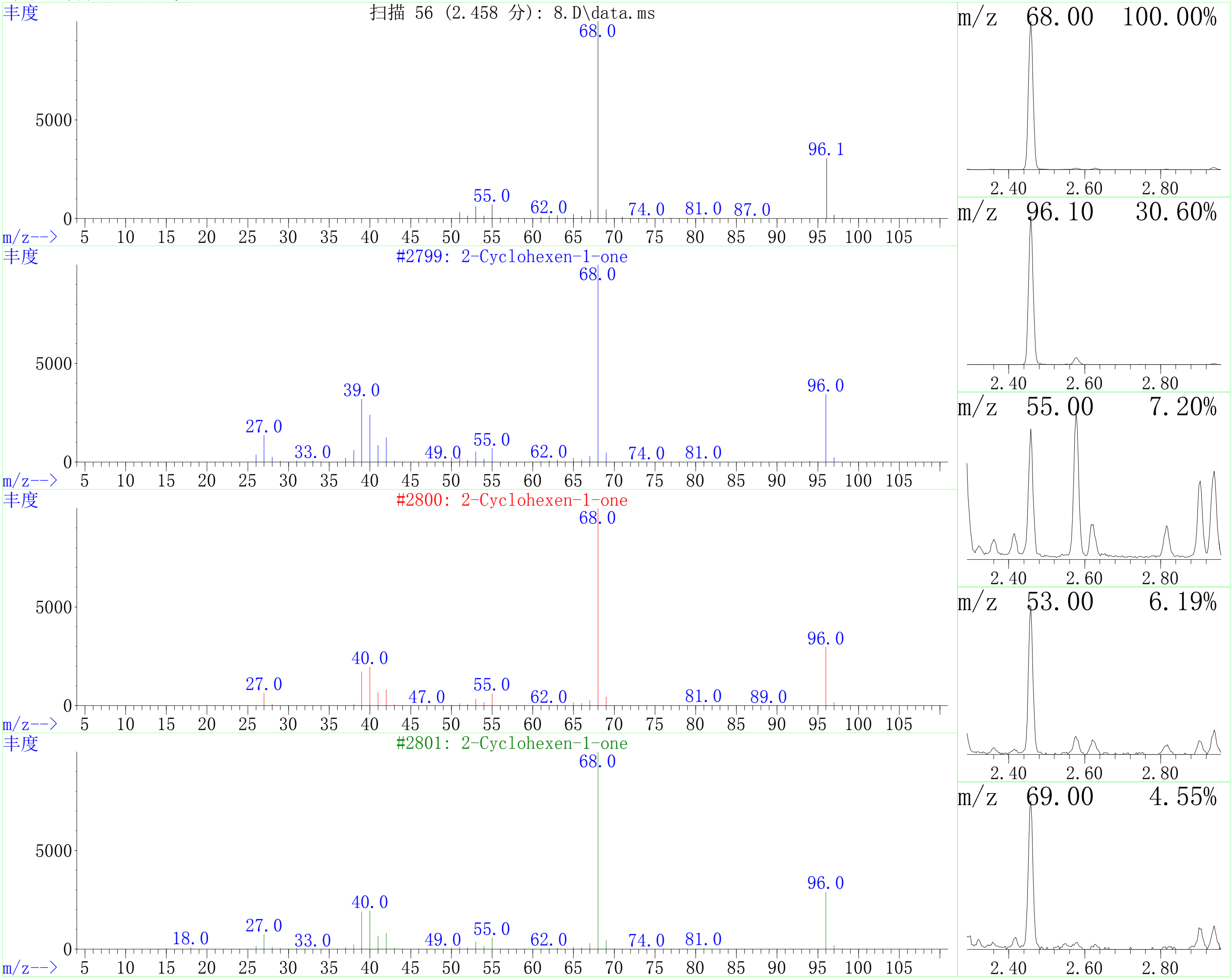

Data File: D:\msdchem\1\data\徐雄发\方岩雄-肖业鹏\8.D
样品: 8

峰编号: 1　　2.458 分钟处　面积: 3973205　面积 % 3.14

每个谱库中 3 个最匹配的记录。　　Ref\#　CAS\#　匹配度

D:\DATABASE\DEMO.L　　未检索到匹配。

C:\DATABASE\NIST11.L

1	2-Cyclohexen-1-one	2799	000930-68-7	90
2	2-Cyclohexen-1-one	2800	000930-68-7	90
3	2-Cyclohexen-1-one	2801	000930-68-7	90

未知谱图基于顶点

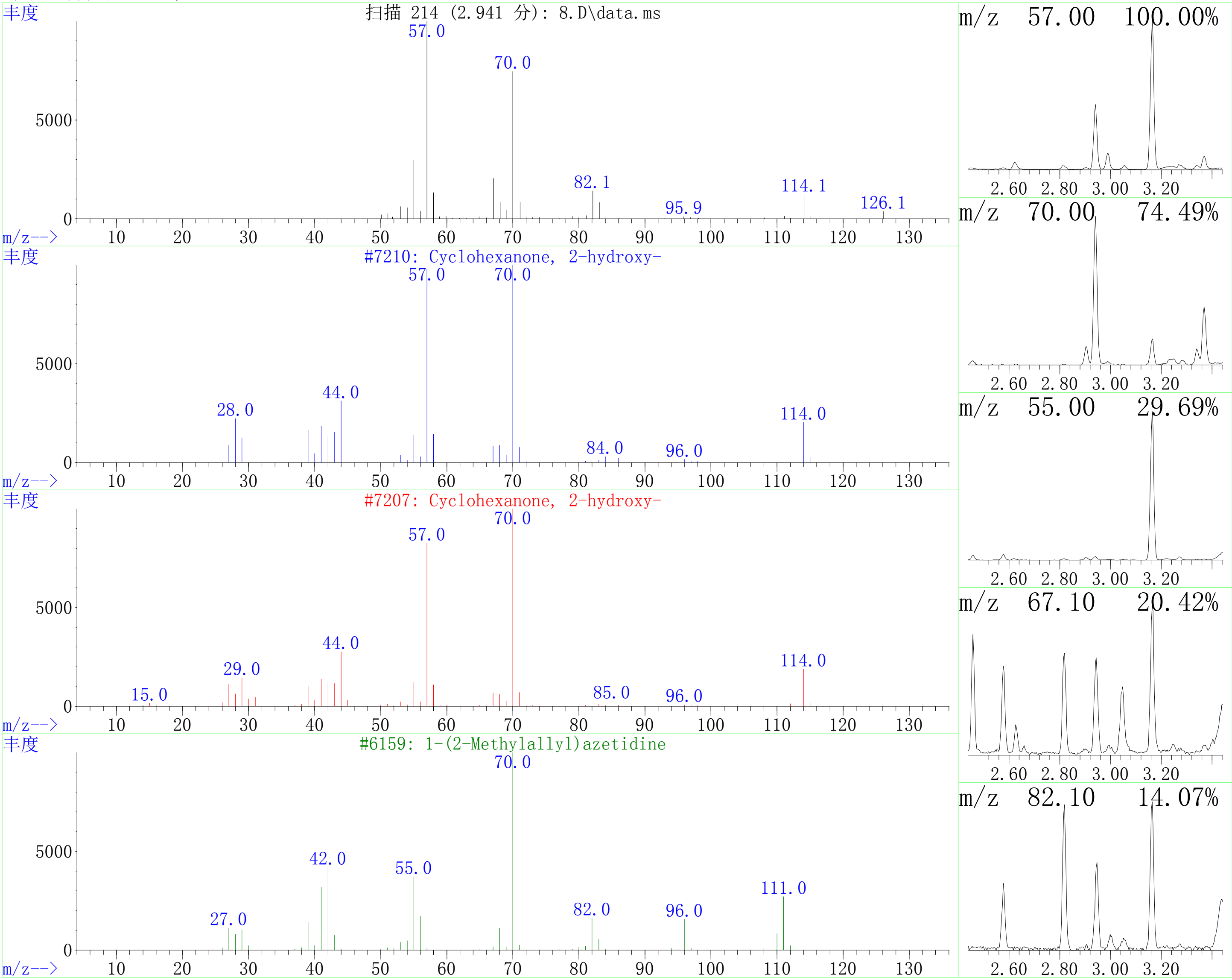

Data File: D:\msdchem\1\data\徐雄发\方岩雄-肖业鹏\8.D
样品: 8

峰编号: 2　　2.941 分钟处　面积:　1456617　面积 % 1.15

每个谱库中 3 个最匹配的记录。　　Ref\#　CAS\#　匹配度

D:\DATABASE\DEMO.L　　未检索到匹配。

C:\DATABASE\NIST11.L

		Ref\#	CAS\#	匹配度
1	Cyclohexanone, 2-hydroxy-	7210	000533-60-8	58
2	Cyclohexanone, 2-hydroxy-	7207	000533-60-8	53
3	1-(2-Methylallyl)azetidine	6159	1000194-99-1	37

未知谱图基于顶点

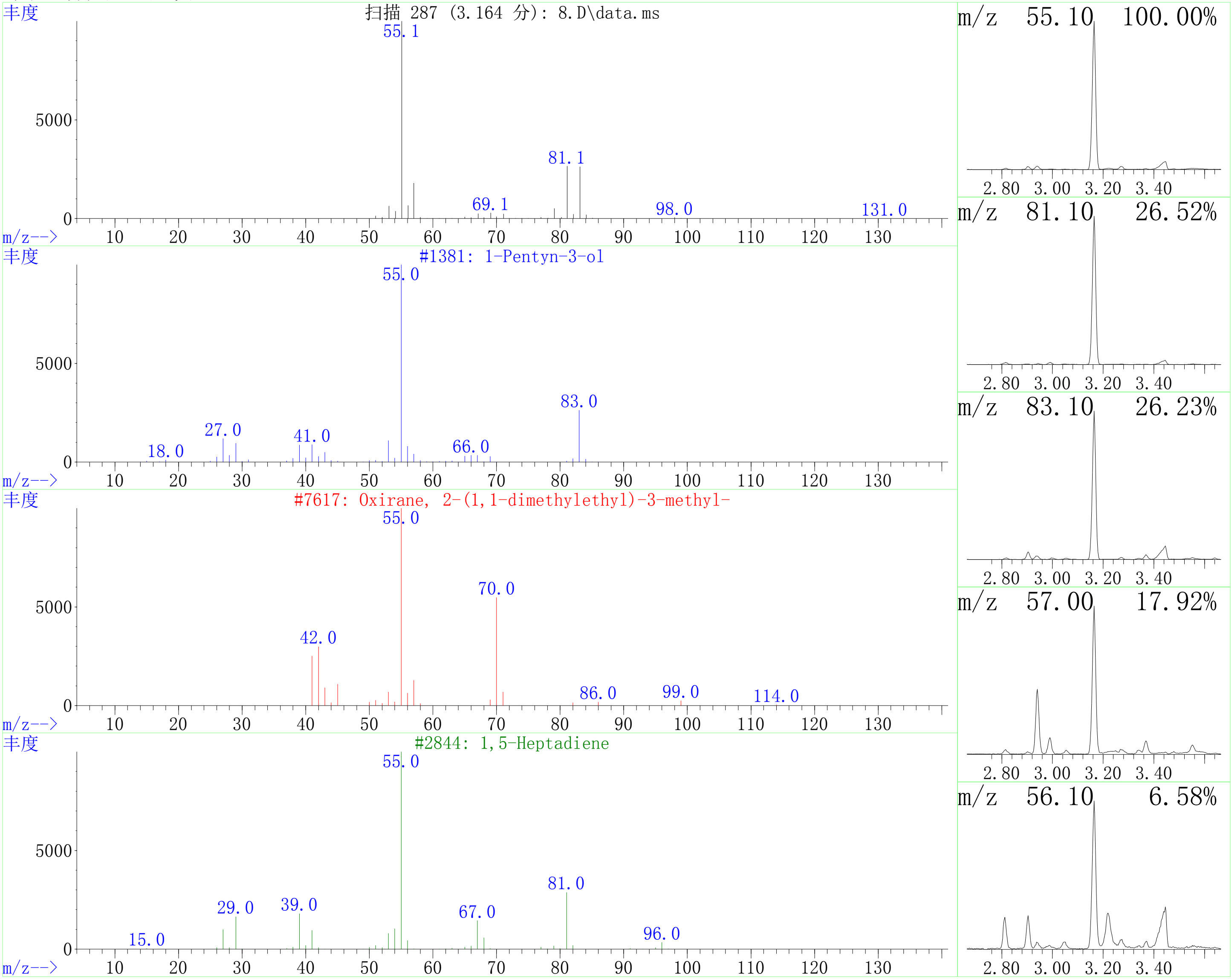

Data File: D:\msdchem\1\data\徐雄发\方岩雄-肖业鹏\8.D
样品: 8

峰编号: 3　　3.164 分钟处　面积: 11489845　面积 % 9.07

每个谱库中 3 个最匹配的记录。　　Ref\#　CAS\#　匹配度

D:\DATABASE\DEMO.L　　未检索到匹配。

C:\DATABASE\NIST11.L

		Ref\#	CAS\#	匹配度
1	1-Pentyn-3-ol	1381	004187-86-4	33
2	Oxirane, 2-(1,1-dimethylethyl)-3...	7617	053897-30-6	23
3	1,5-Heptadiene	2844	001541-23-7	17

未知谱图基于顶点

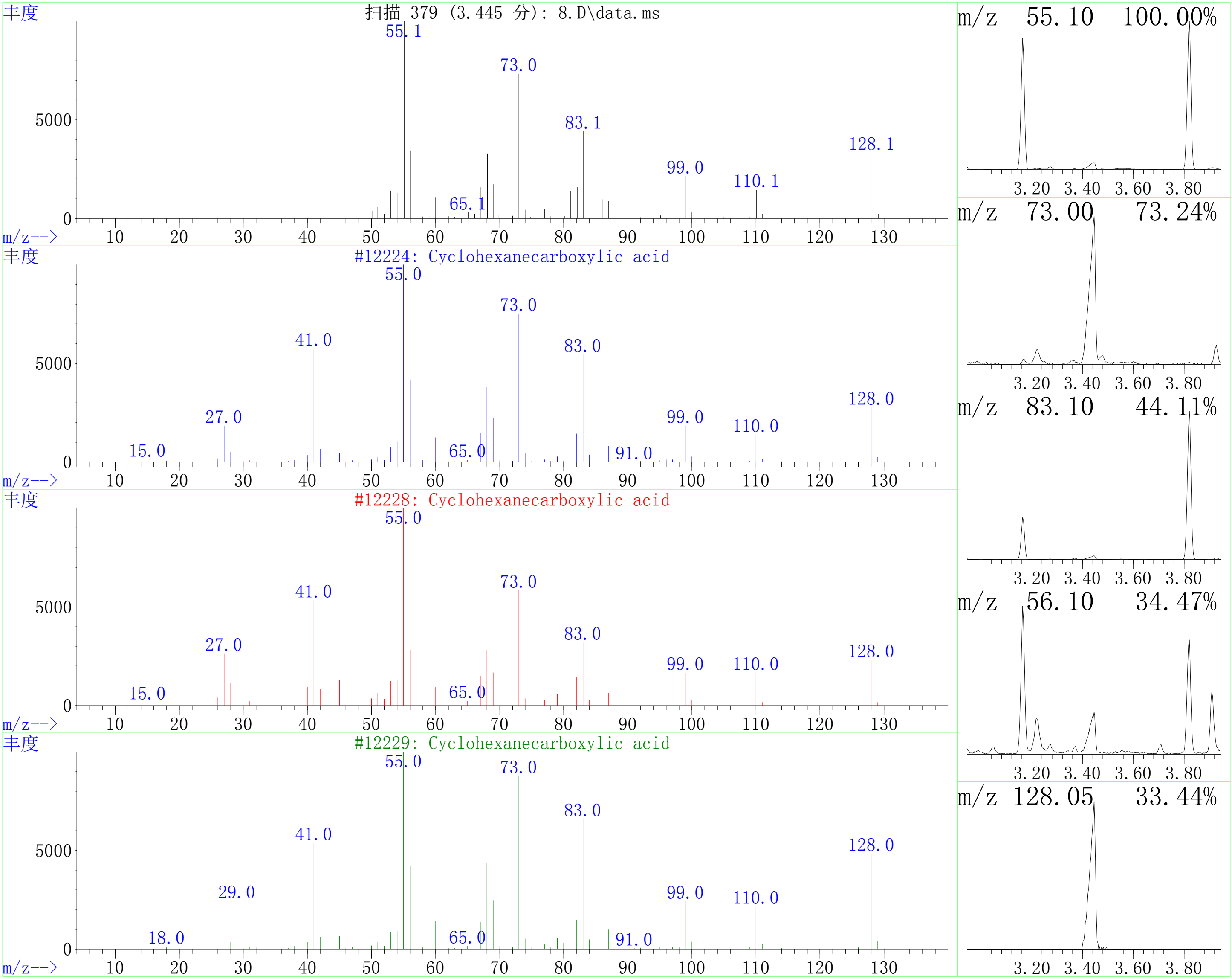

Data File: D:\msdchem\1\data\徐雄发\方岩雄-肖业鹏\8.D
样品: 8

峰编号: 4 3.445 分钟处 面积: 2700186 面积 % 2.13

每个谱库中 3 个最匹配的记录。 Ref\# CAS\# 匹配度

D:\DATABASE\DEMO.L 未检索到匹配。

C:\DATABASE\NIST11.L

		Ref\#	CAS\#	匹配度
1	Cyclohexanecarboxylic acid	12224	000098-89-5	97
2	Cyclohexanecarboxylic acid	12228	000098-89-5	91
3	Cyclohexanecarboxylic acid	12229	000098-89-5	87

未知谱图基于顶点

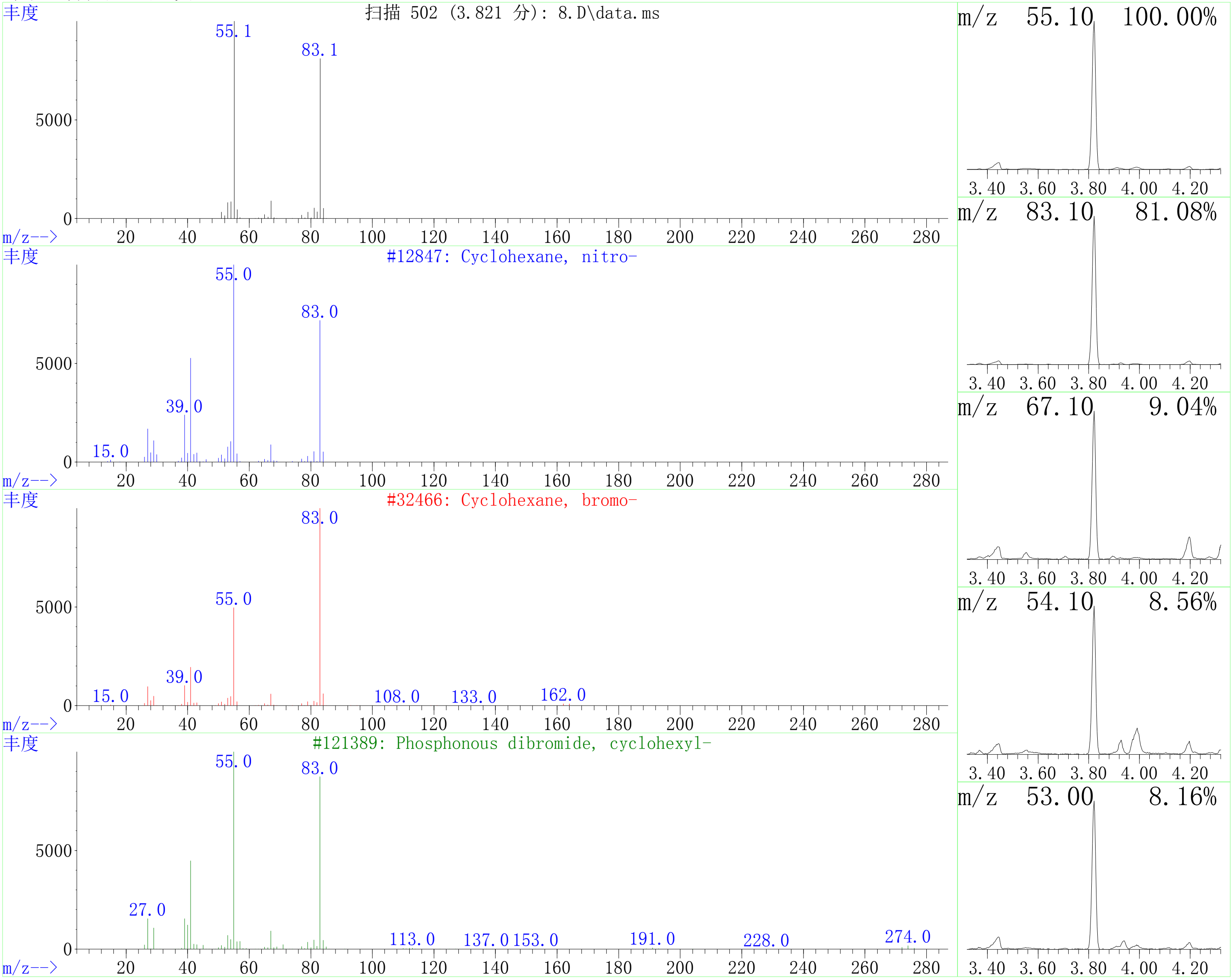

Data File: D:\msdchem\1\data\徐雄发\方岩雄-肖业鹏\8.D
样品: 8

峰编号: 5   3.821 分钟处 面积: 15650168 面积 % 12.36

每个谱库中 3 个最匹配的记录。	Ref\#	CAS\#	匹配度
D:\DATABASE\DEMO.L 未检索到匹配。			
C:\DATABASE\NIST11.L			
1 Cyclohexane, nitro-	12847	001122-60-7	83
2 Cyclohexane, bromo-	32466	000108-85-0	83
3 Phosphonous dibromide, cyclohexyl-	121389	086012-32-0	74

未知谱图基于顶点

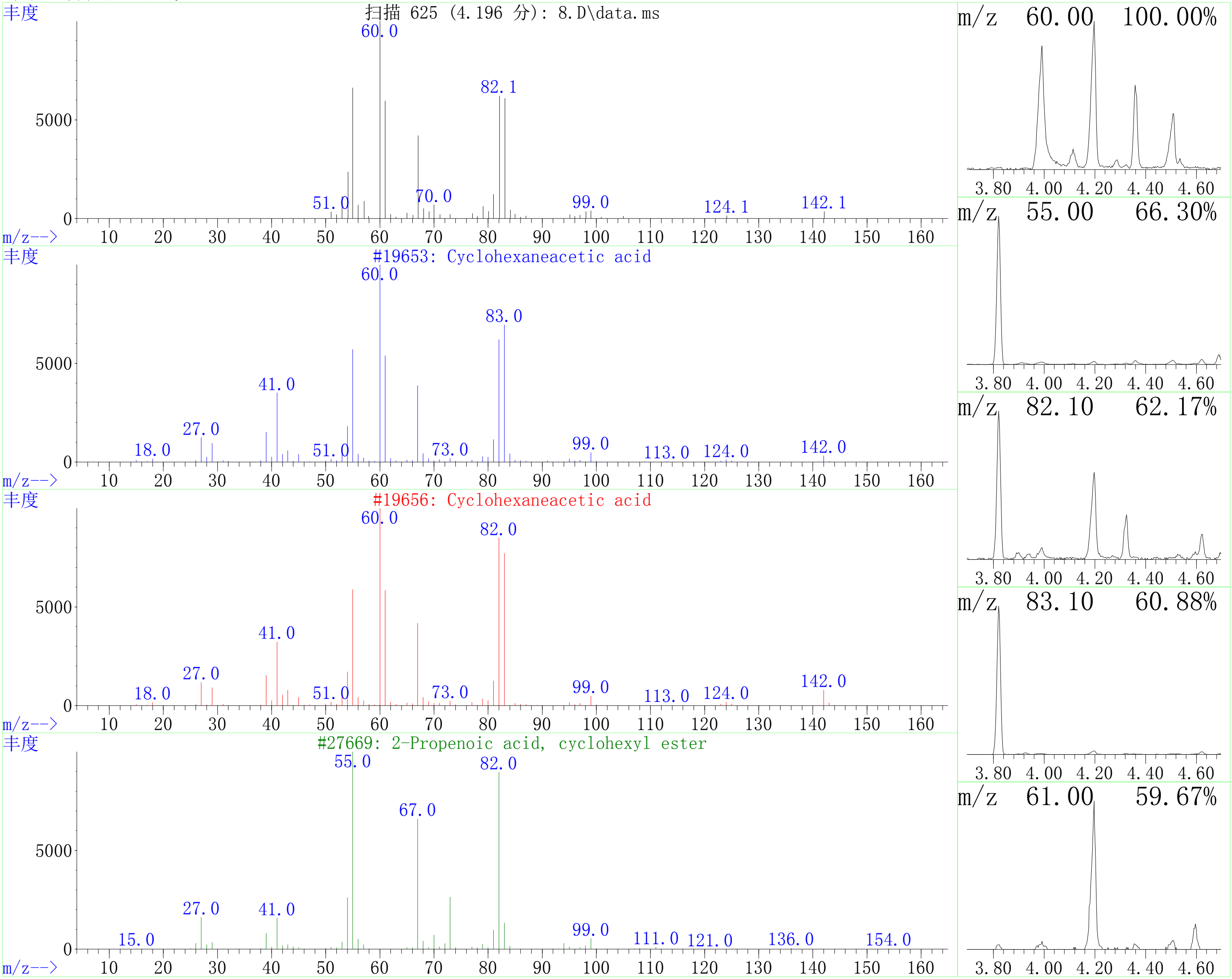

Data File: D:\msdchem\1\data\徐雄发\方岩雄-肖业鹏\8.D
样品: 8

峰编号: 6 4.196 分钟处 面积: 1457115 面积 % 1.15

每个谱库中 3 个最匹配的记录。	Ref\#	CAS\#	匹配度
D:\DATABASE\DEMO.L 未检索到匹配。			
C:\DATABASE\NIST11.L			
1 Cyclohexaneacetic acid	19653	005292-21-7	97
2 Cyclohexaneacetic acid	19656	005292-21-7	78
3 2-Propenoic acid, cyclohexyl ester	27669	003066-71-5	27

未知谱图基于顶点

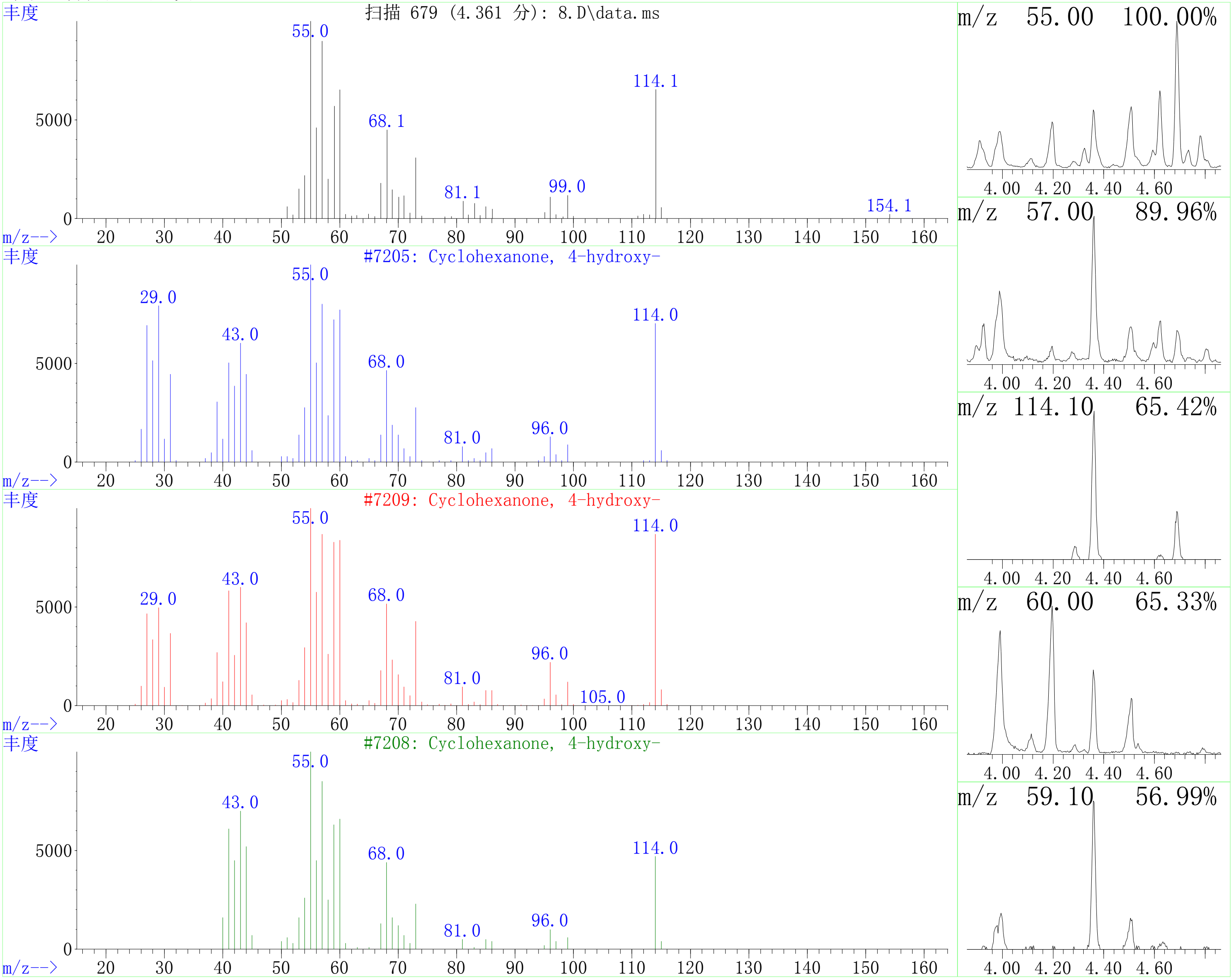

Data File: D:\msdchem\1\data\徐雄发\方岩雄-肖业鹏\8.D
样品: 8

峰编号: 7 4.361 分钟处 面积: 1464243 面积 % 1.16

每个谱库中 3 个最匹配的记录。 Ref\# CAS\# 匹配度

D:\DATABASE\DEMO.L 未检索到匹配。

C:\DATABASE\NIST11.L

		Ref\#	CAS\#	匹配度
1	Cyclohexanone, 4-hydroxy-	7205	013482-22-9	97
2	Cyclohexanone, 4-hydroxy-	7209	013482-22-9	80
3	Cyclohexanone, 4-hydroxy-	7208	013482-22-9	64

未知谱图基于顶点

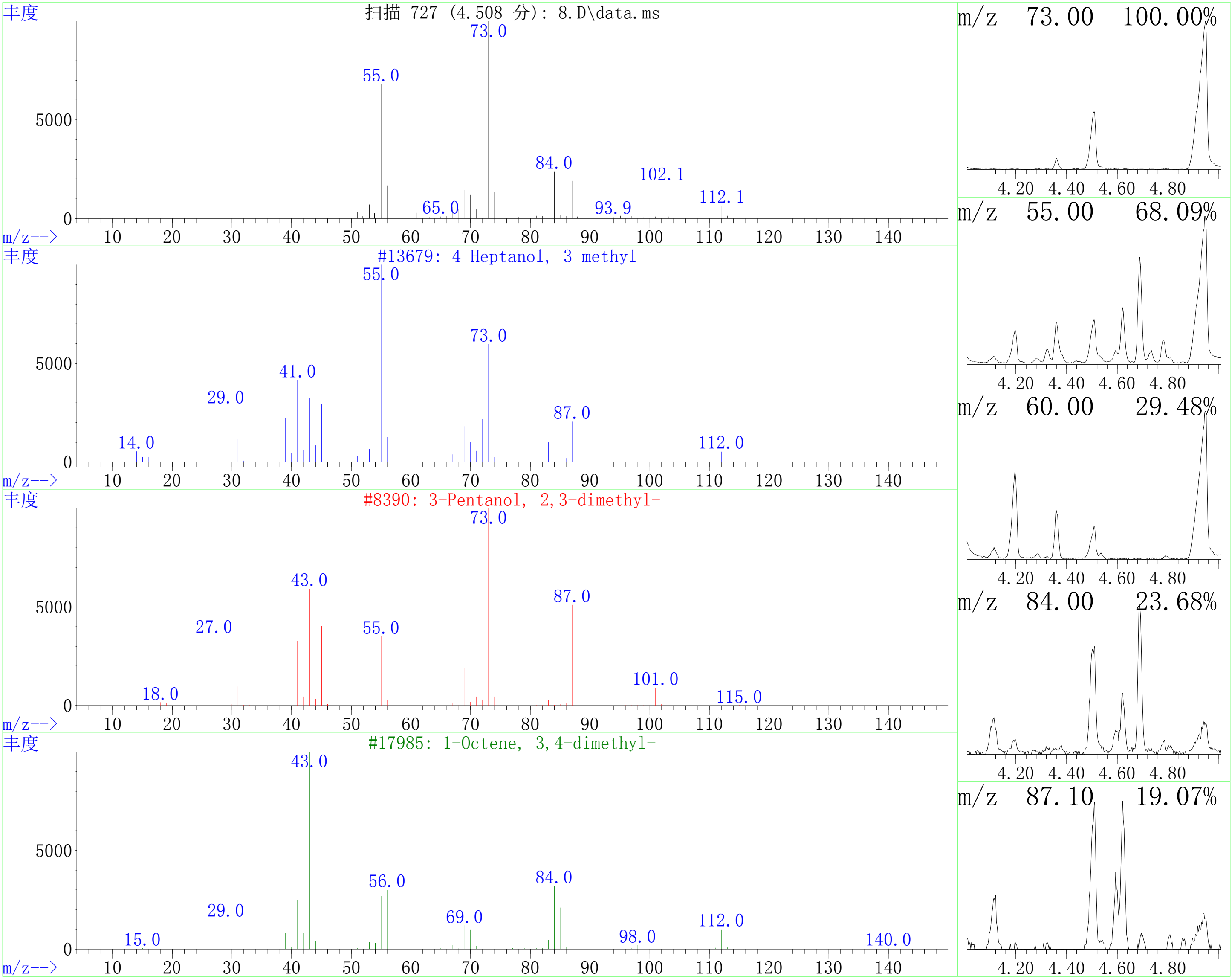

Data File: D:\msdchem\1\data\徐雄发\方岩雄-肖业鹏\8.D
样品: 8

峰编号: 8 4.508 分钟处 面积: 1741486 面积 % 1.38

每个谱库中 3 个最匹配的记录。 Ref\# CAS\# 匹配度

D:\DATABASE\DEMO.L 未检索到匹配。

C:\DATABASE\NIST11.L

		Ref\#	CAS\#	匹配度
1	4-Heptanol, 3-methyl-	13679	001838-73-9	38
2	3-Pentanol, 2,3-dimethyl-	8390	000595-41-5	35
3	1-Octene, 3,4-dimethyl-	17985	056728-11-1	22

未知谱图基于顶点

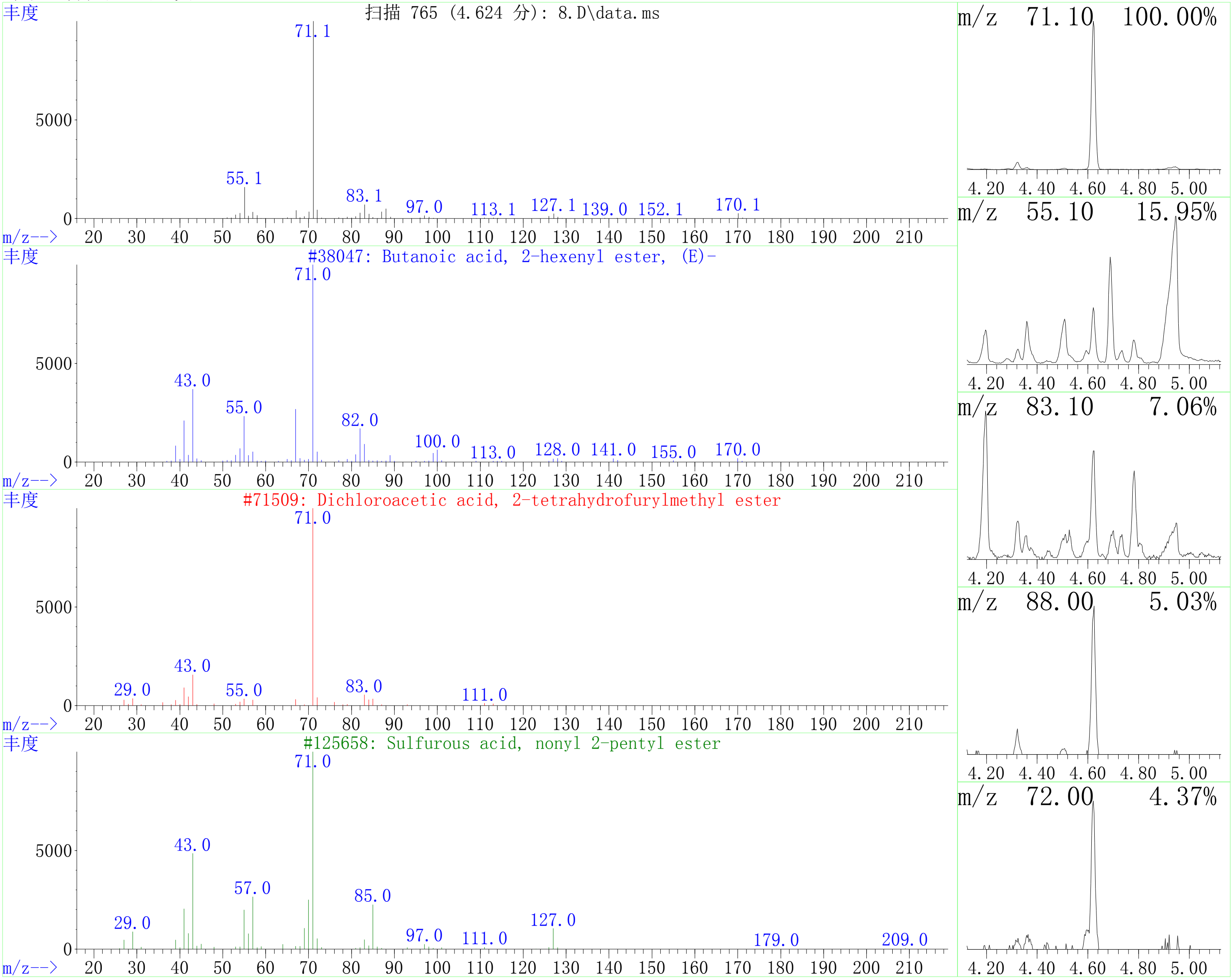

Data File: D:\msdchem\1\data\徐雄发\方岩雄-肖业鹏\8.D
样品: 8

峰编号: 9 4.624 分钟处 面积: 2774519 面积 % 2.19

每个谱库中 3 个最匹配的记录。 Ref\# CAS\# 匹配度

D:\DATABASE\DEMO.L 未检索到匹配。

C:\DATABASE\NIST11.L

		Ref\#	CAS\#	匹配度
1	Butanoic acid, 2-hexenyl ester, ...	38047	053398-83-7	39
2	Dichloroacetic acid, 2-tetrahydr...	71509	004697-00-1	39
3	Sulfurous acid, nonyl 2-pentyl e...	125658	1000309-15-8	39

未知谱图基于顶点

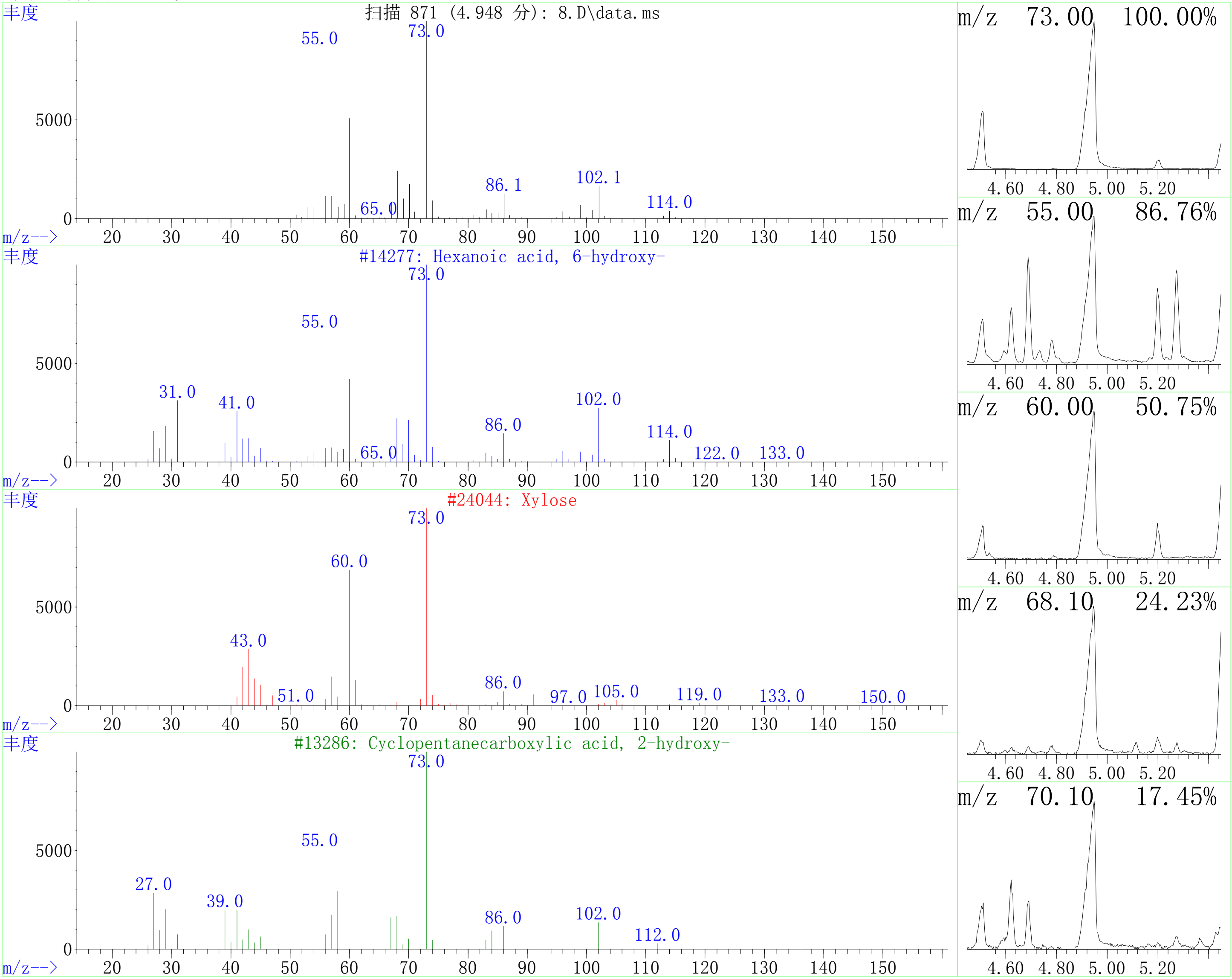

Data File: D:\msdchem\1\data\徐雄发\方岩雄-肖业鹏\8.D
样品: 8

峰编号: 10 4.948 分钟处 面积: 6431080 面积 % 5.08

每个谱库中 3 个最匹配的记录。	Ref\#	CAS\#	匹配度
D:\DATABASE\DEMO.L 未检索到匹配。			
C:\DATABASE\NIST11.L			
1 Hexanoic acid, 6-hydroxy-	14277	001191-25-9	53
2 Xylose	24044	000058-86-6	32
3 Cyclopentanecarboxylic acid, 2-h...	13286	081887-89-0	32

未知谱图基于顶点

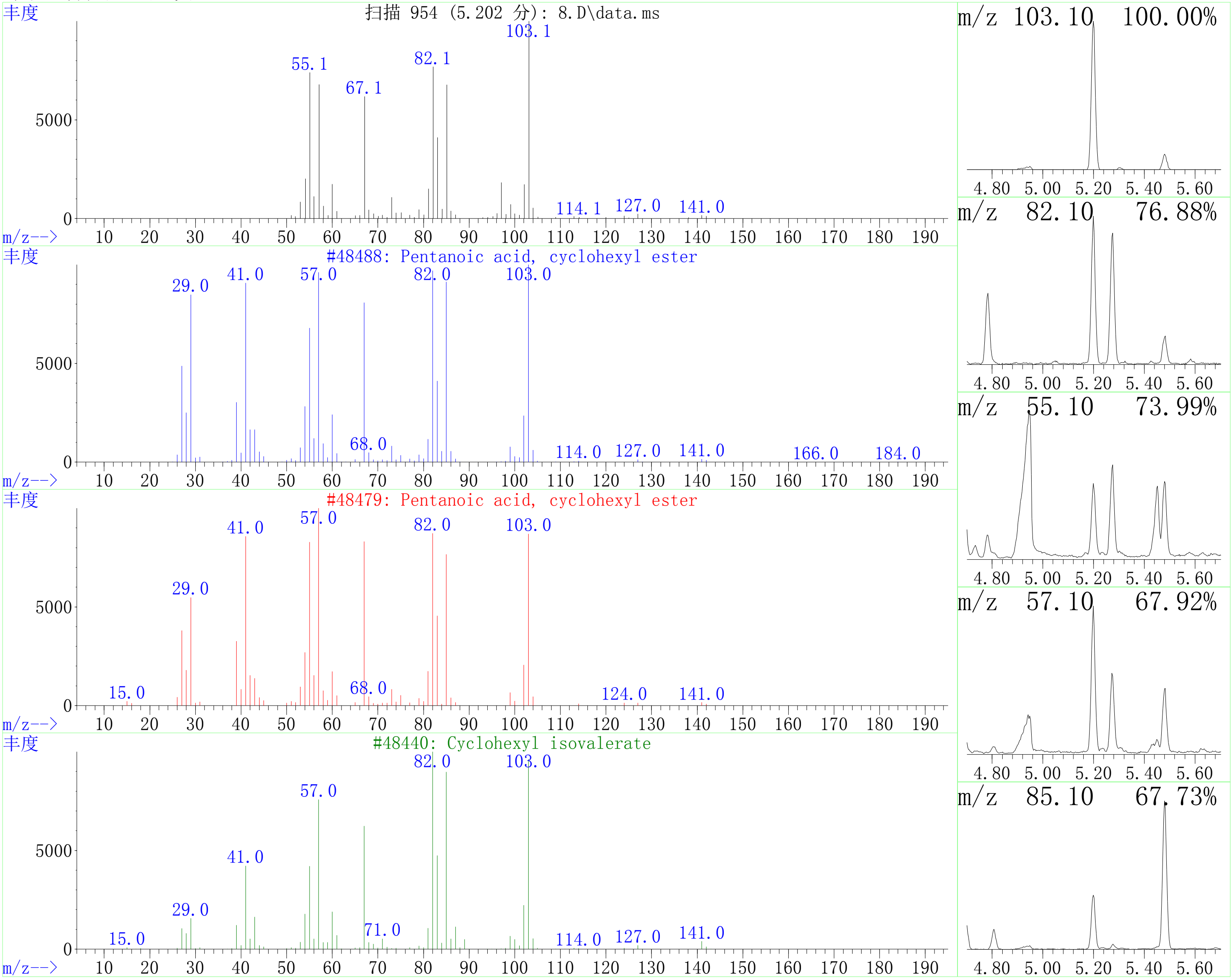

Data File: D:\msdchem\1\data\徐雄发\方岩雄-肖业鹏\8.D
样品: 8

峰编号: 11 5.202 分钟处 面积: 3273767 面积 % 2.59

每个谱库中 3 个最匹配的记录。	Ref\#	CAS\#	匹配度
D:\DATABASE\DEMO.L 未检索到匹配。			
C:\DATABASE\NIST11.L			
1 Pentanoic acid, cyclohexyl ester	48488	001551-43-5	87
2 Pentanoic acid, cyclohexyl ester	48479	001551-43-5	81
3 Cyclohexyl isovalerate	48440	007774-44-9	80

未知谱图基于顶点

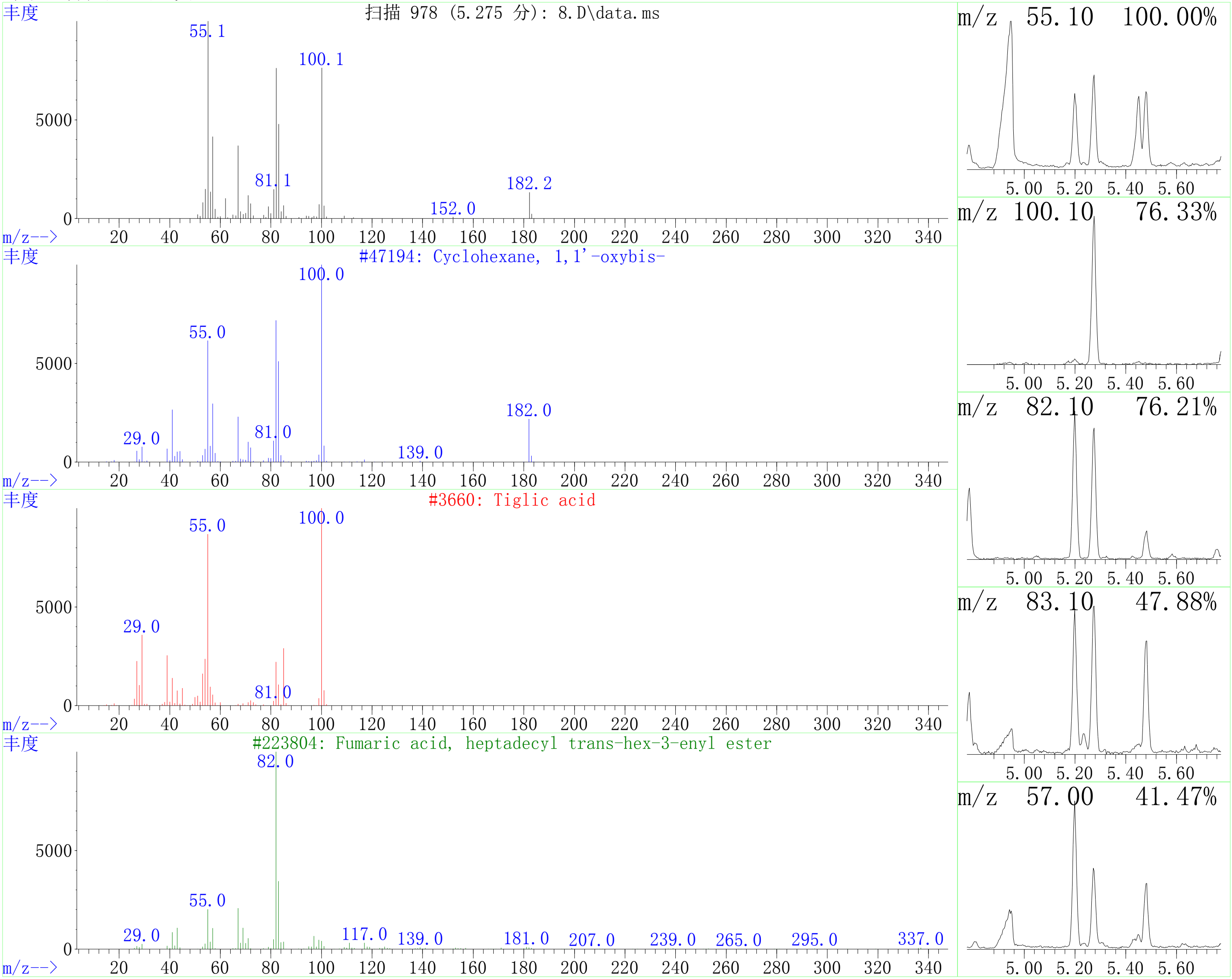

Data File: D:\msdchem\1\data\徐雄发\方岩雄-肖业鹏\8.D
样品: 8

峰编号: 12 5.275 分钟处 面积: 2493456 面积 % 1.97

每个谱库中 3 个最匹配的记录。	Ref\#	CAS\#	匹配度
D:\DATABASE\DEMO.L 未检索到匹配。			
C:\DATABASE\NIST11.L			
1 Cyclohexane, 1,1'-oxybis-	47194	004645-15-2	93
2 Tiglic acid	3660	000080-59-1	38
3 Fumaric acid, heptadecyl trans-h...	223804	1000348-89-7	35

未知谱图基于顶点

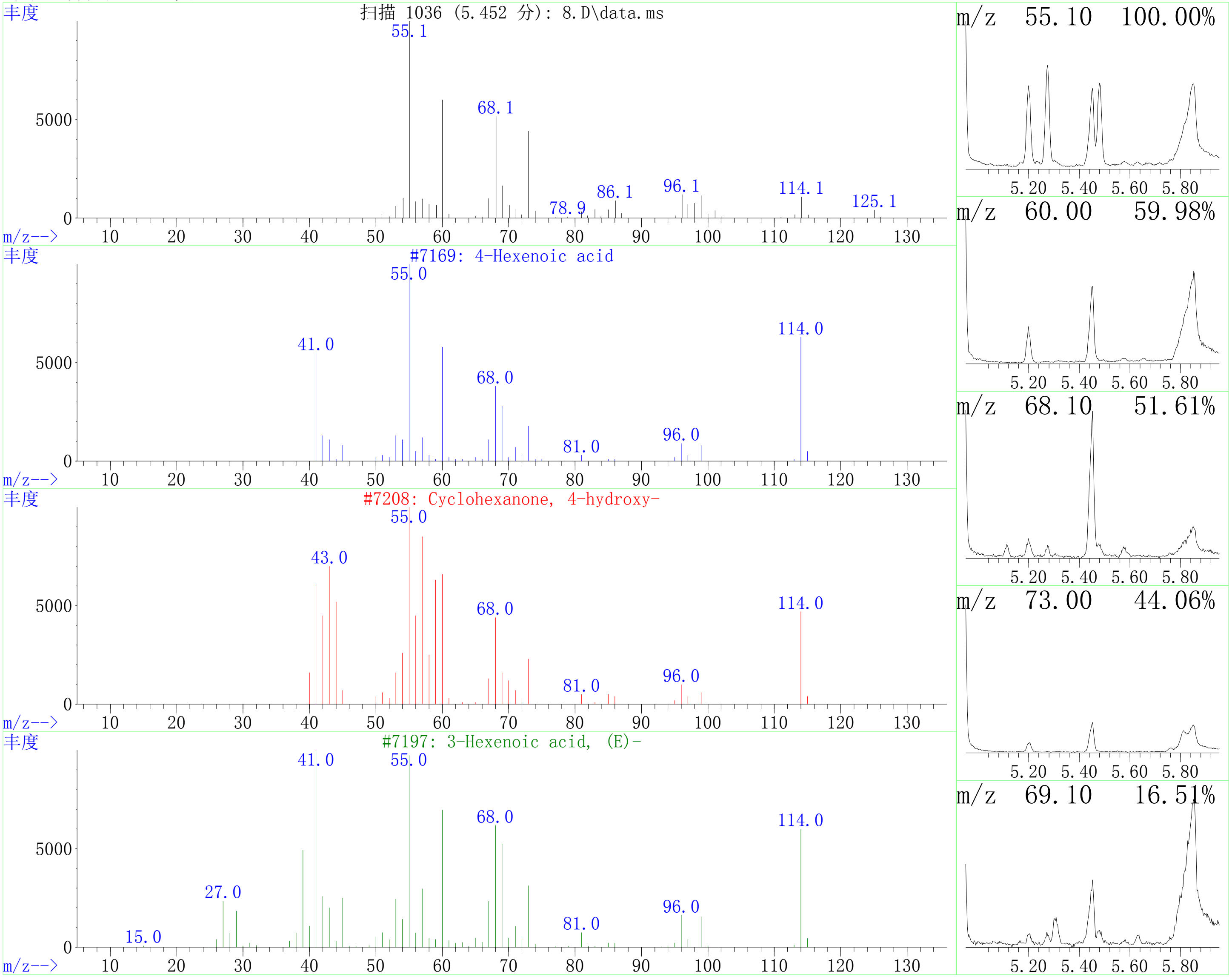

Data File: D:\msdchem\1\data\徐雄发\方岩雄-肖业鹏\8.D
样品: 8

峰编号: 13　　5.452 分钟处　面积: 1610644　面积 % 1.27

每个谱库中 3 个最匹配的记录。　Ref\#　CAS\#　匹配度

D:\DATABASE\DEMO.L　未检索到匹配。

C:\DATABASE\NIST11.L

		Ref\#	CAS\#	匹配度
1	4-Hexenoic acid	7169	035194-36-6	83
2	Cyclohexanone, 4-hydroxy-	7208	013482-22-9	64
3	3-Hexenoic acid, (E)-	7197	001577-18-0	64

谱库检索报告 - 化学工作站积分器

未知谱图基于顶点

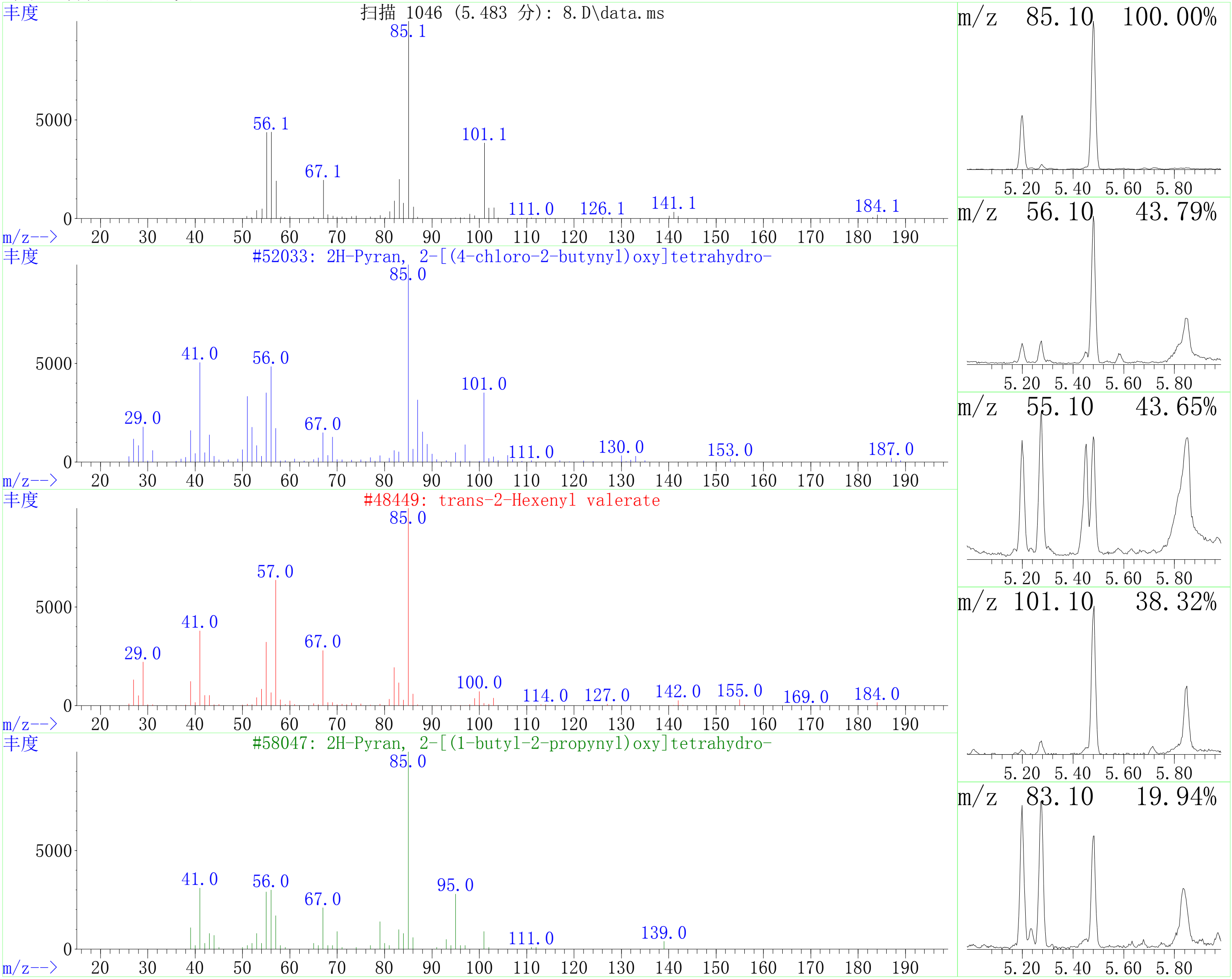

Data File: D:\msdchem\1\data\徐雄发\方岩雄-肖业鹏\8.D
样品: 8

峰编号: 14　　5.483 分钟处　面积: 2943356　面积 % 2.32

每个谱库中 3 个最匹配的记录。	Ref\#	CAS\#	匹配度
D:\DATABASE\DEMO.L　未检索到匹配。			
C:\DATABASE\NIST11.L			
1 2H-Pyran, 2-[(4-chloro-2-butynyl...	52033	067727-01-9	50
2 trans-2-Hexenyl valerate	48449	056922-74-8	43
3 2H-Pyran, 2-[(1-butyl-2-propynyl...	58047	000831-83-4	42

谱库检索报告 - 化学工作站积分器

未知谱图基于顶点

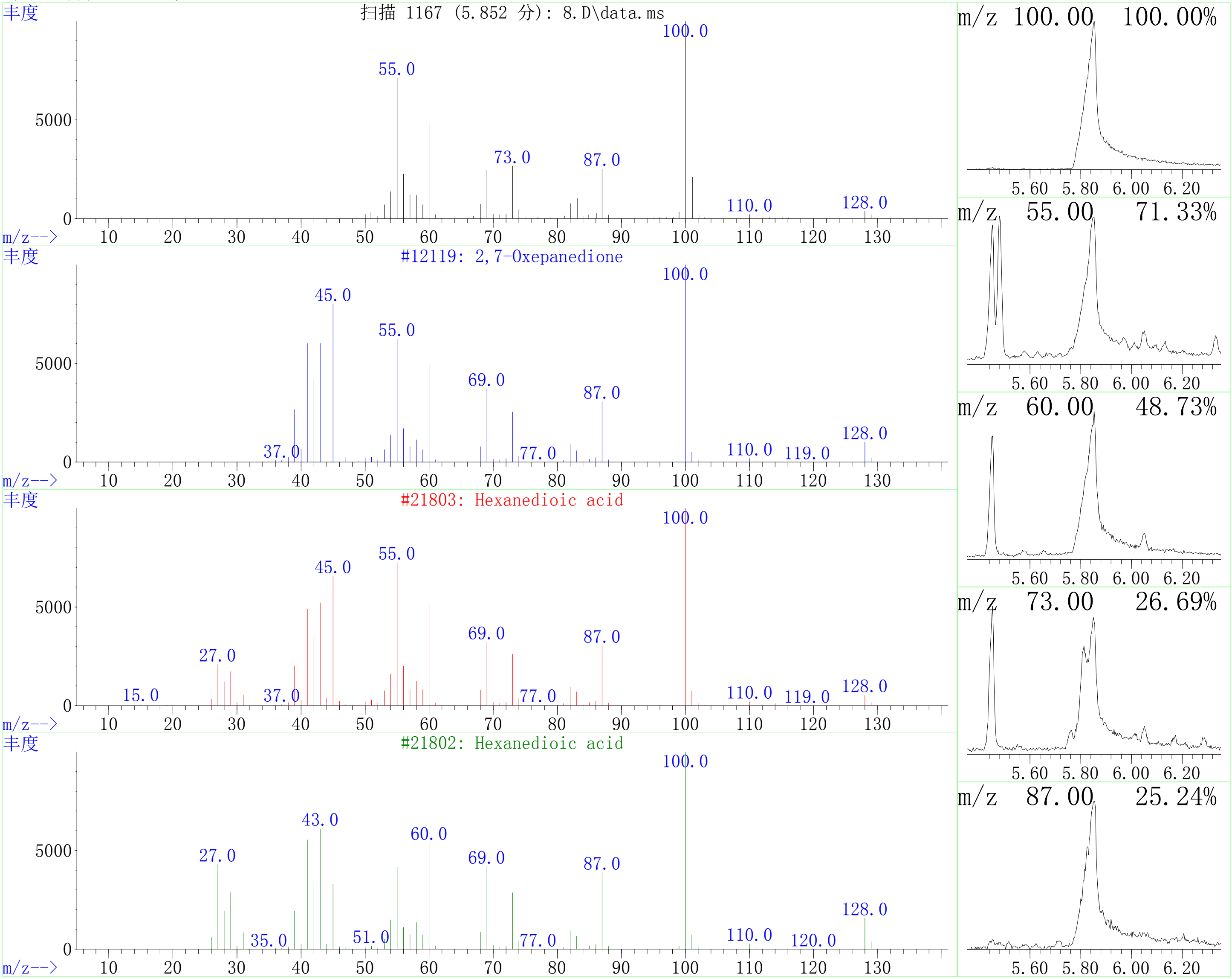

Data File: D:\msdchem\1\data\徐雄发\方岩雄-肖业鹏\8.D
样品: 8

峰编号: 15　　5.852 分钟处　面积: 　6158234　面积 % 4.86

每个谱库中 3 个最匹配的记录。	Ref\#	CAS\#	匹配度
D:\DATABASE\DEMO.L　　未检索到匹配。			
C:\DATABASE\NIST11.L			
1 2,7-Oxepanedione	12119	002035-75-8	64
2 Hexanedioic acid	21803	000124-04-9	50
3 Hexanedioic acid	21802	000124-04-9	42

未知谱图基于顶点

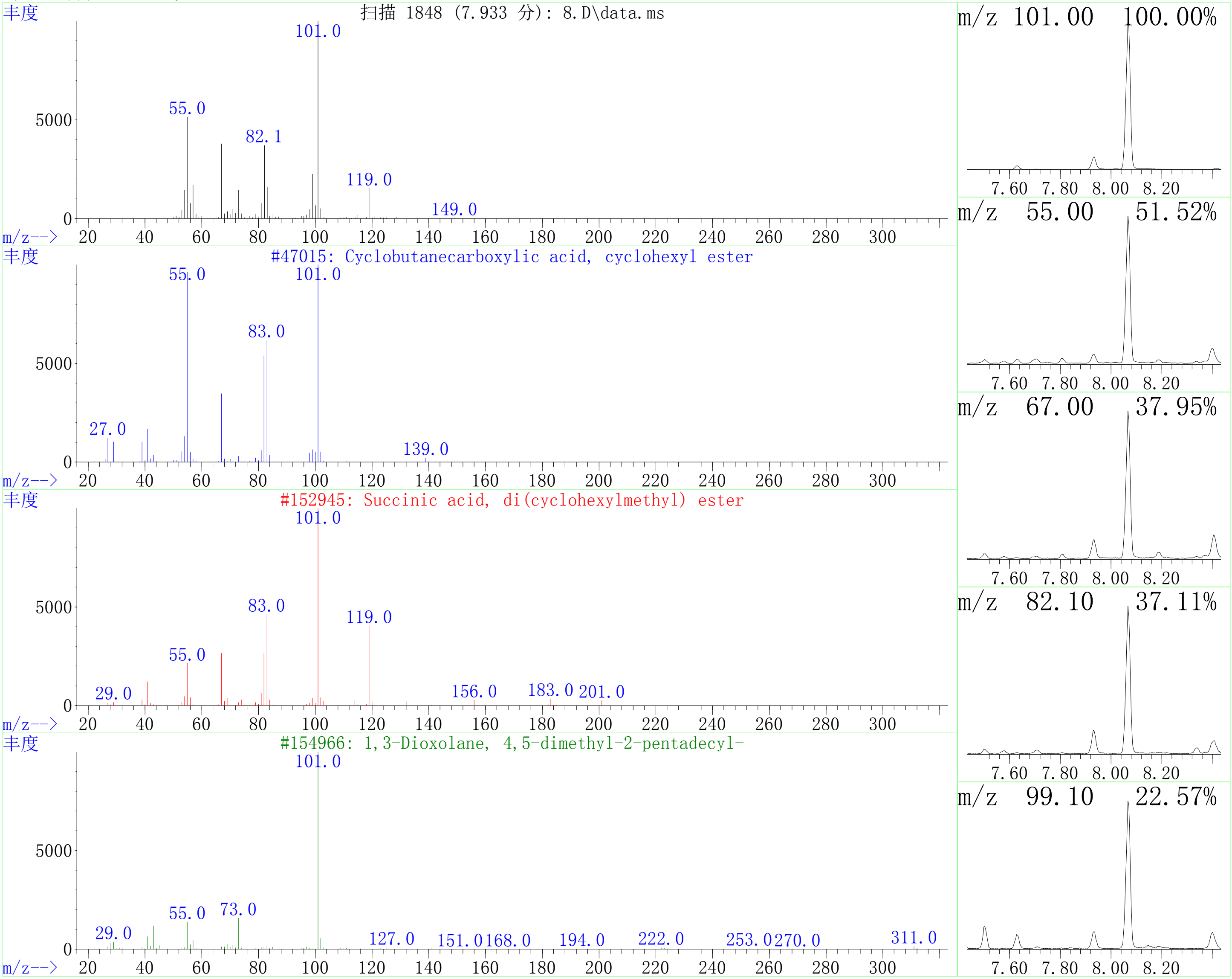

Data File: D:\msdchem\1\data\徐雄发\方岩雄-肖业鹏\8.D
样品: 8

峰编号: 16 7.933 分钟处 面积: 2045648 面积 % 1.62

每个谱库中 3 个最匹配的记录。 Ref\# CAS\# 匹配度

D:\DATABASE\DEMO.L 未检索到匹配。

C:\DATABASE\NIST11.L

		Ref\#	CAS\#	匹配度
1	Cyclobutanecarboxylic acid, cycl...	47015	1000282-60-7	53
2	Succinic acid, di(cyclohexylmeth...	152945	1000325-83-4	45
3	1,3-Dioxolane, 4,5-dimethyl-2-pe...	154966	056599-61-2	43

未知谱图基于顶点

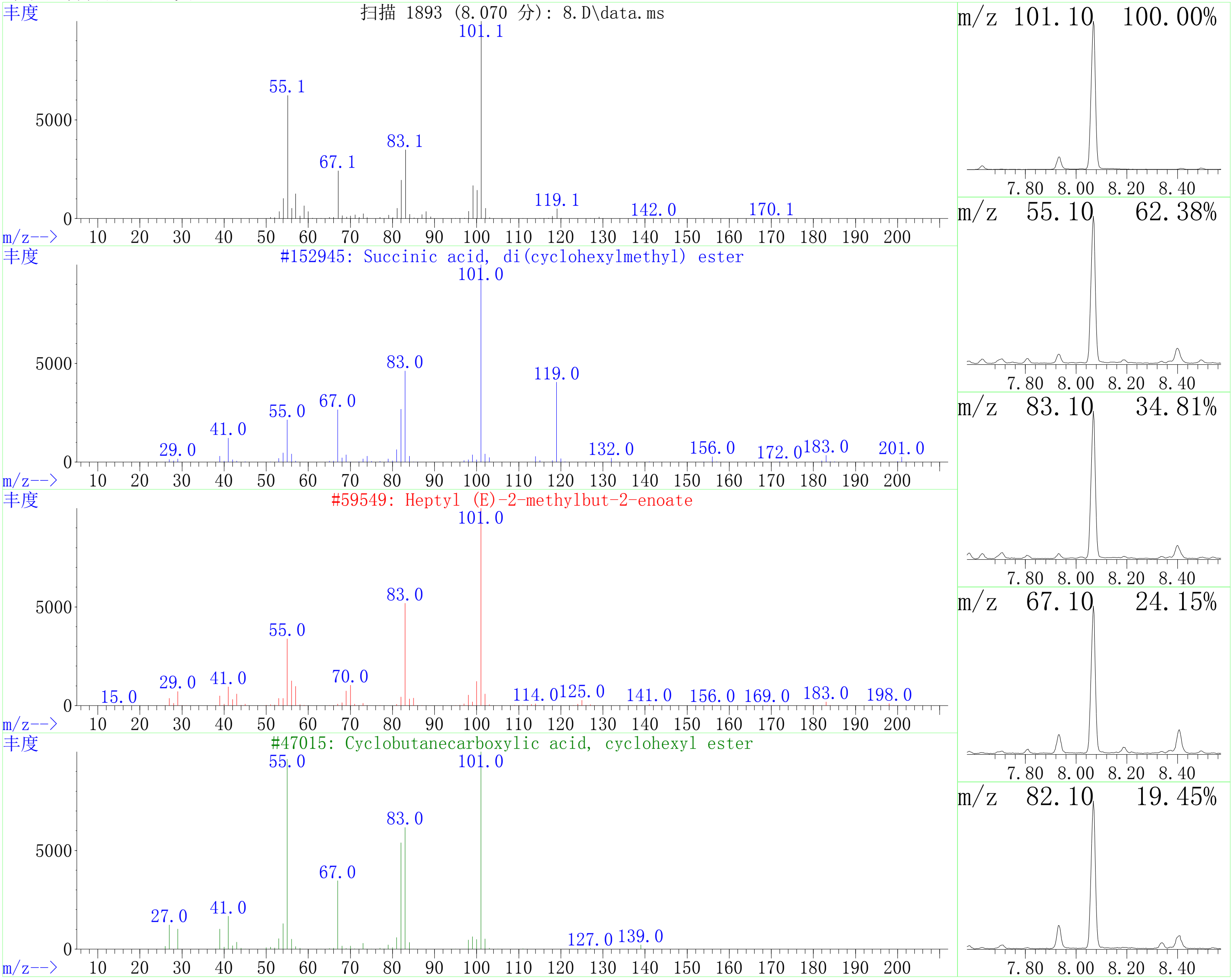

Data File: D:\msdchem\1\data\徐雄发\方岩雄-肖业鹏\8.D
样品: 8

峰编号: 17 8.070 分钟处 面积: 23482636 面积 % 18.54

每个谱库中 3 个最匹配的记录。 Ref\# CAS\# 匹配度

D:\DATABASE\DEMO.L 未检索到匹配。

C:\DATABASE\NIST11.L

		Ref\#	CAS\#	匹配度
1	Succinic acid, di(cyclohexylmeth...	152945	1000325-83-4	64
2	Heptyl (E)-2-methylbut-2-enoate	59549	1000373-73-8	42
3	Cyclobutanecarboxylic acid, cycl...	47015	1000282-60-7	37

未知谱图基于顶点

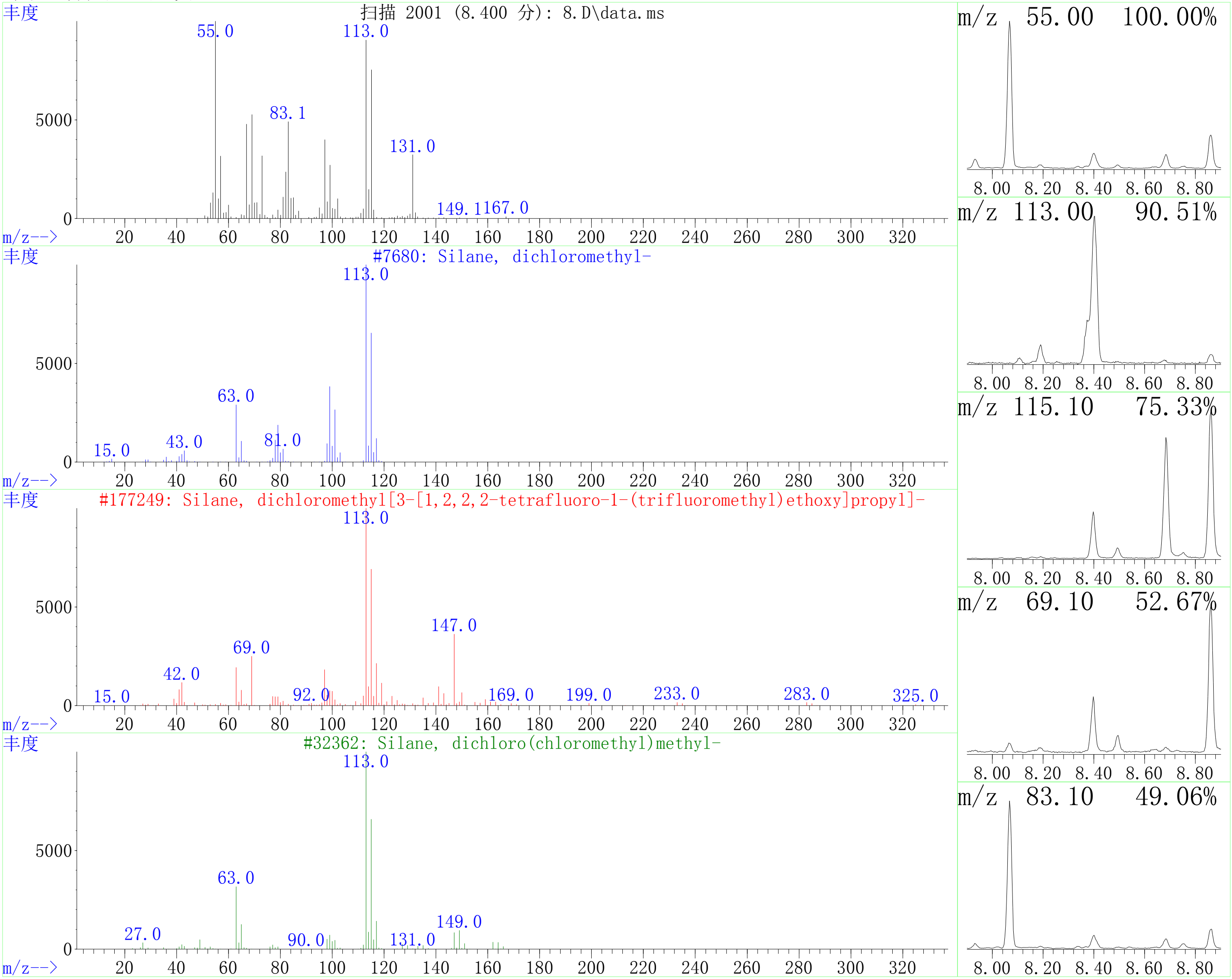

Data File: D:\msdchem\1\data\徐雄发\方岩雄-肖业鹏\8.D
样品: 8

峰编号: 18 8.400 分钟处 面积: 5020177 面积 % 3.96

每个谱库中 3 个最匹配的记录。 Ref\# CAS\# 匹配度

D:\DATABASE\DEMO.L 未检索到匹配。

C:\DATABASE\NIST11.L

		Ref\#	CAS\#	匹配度
1	Silane, dichloromethyl-	7680	000075-54-7	38
2	Silane, dichloromethyl[3-[1,2,2,...	177249	020006-68-2	38
3	Silane, dichloro(chloromethyl)me...	32362	001558-33-4	35

未知谱图基于顶点

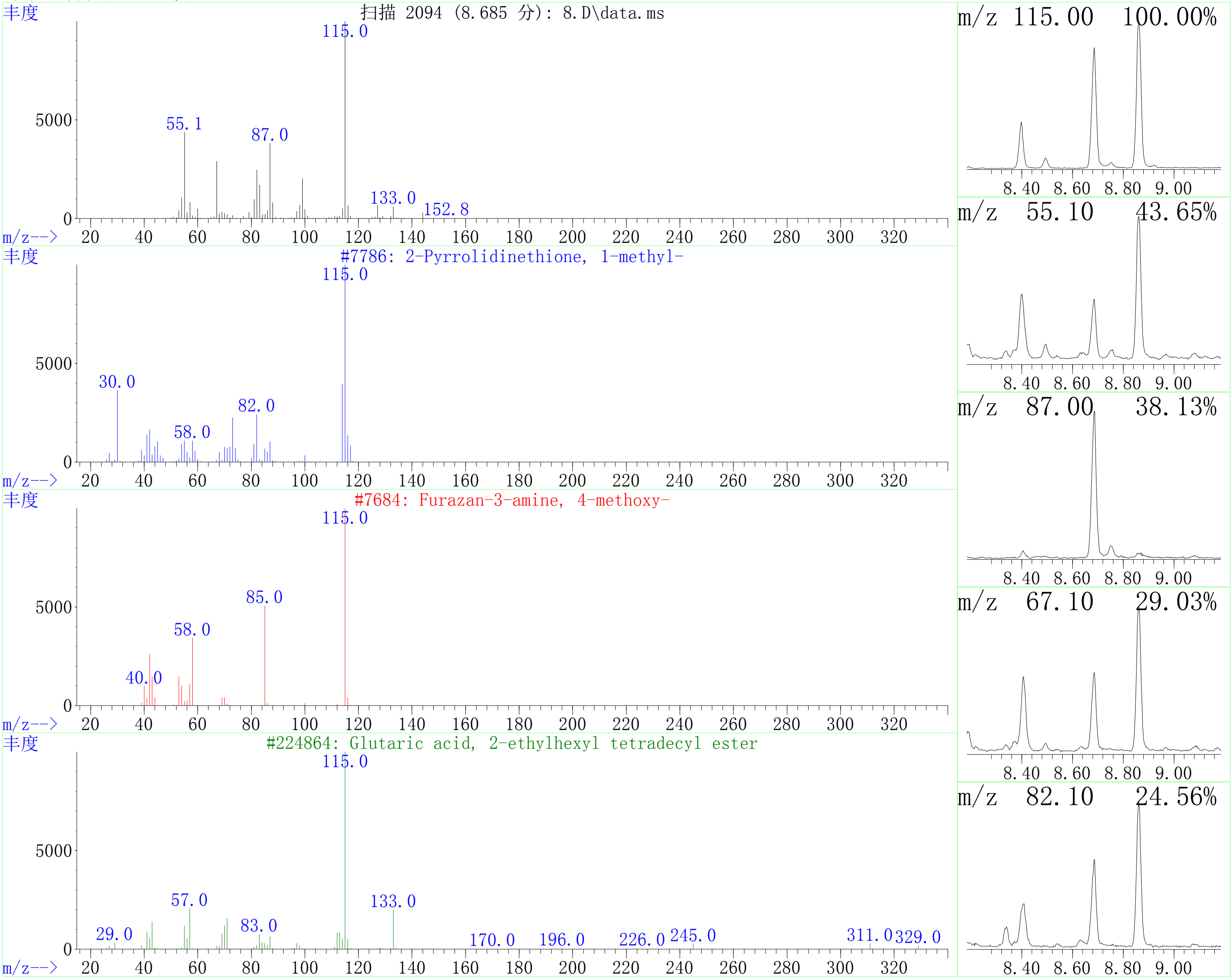

Data File: D:\msdchem\1\data\徐雄发\方岩雄-肖业鹏\8.D
样品: 8

峰编号: 19 8.685 分钟处 面积: 4041965 面积 % 3.19

每个谱库中 3 个最匹配的记录。	Ref\#	CAS\#	匹配度
D:\DATABASE\DEMO.L 未检索到匹配。			
C:\DATABASE\NIST11.L			
1 2-Pyrrolidinethione, 1-methyl-	7786	010441-57-3	32
2 Furazan-3-amine, 4-methoxy-	7684	078350-48-8	27
3 Glutaric acid, 2-ethylhexyl tetr...	224864	1000358-24-6	25

未知谱图基于顶点

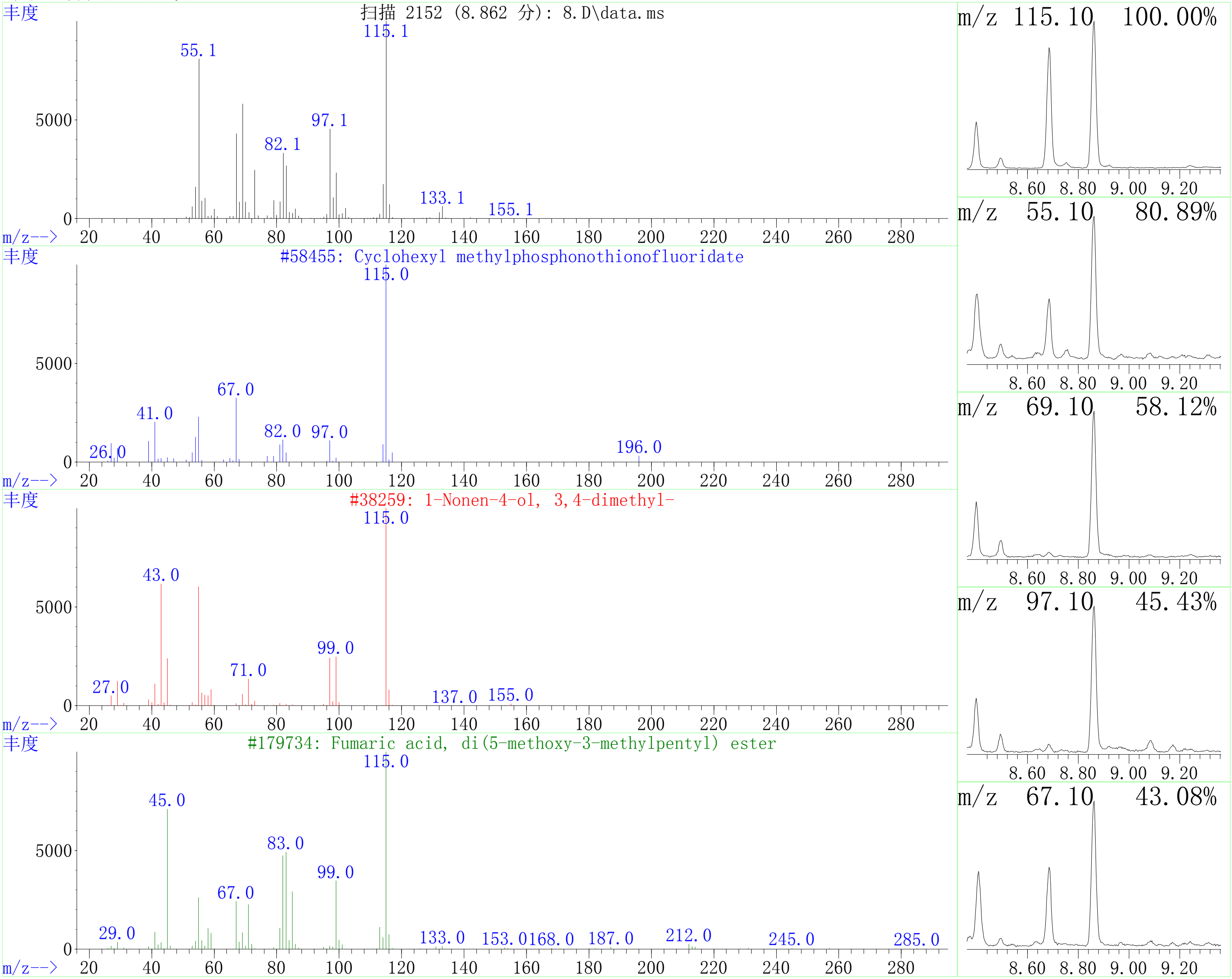

Data File: D:\msdchem\1\data\徐雄发\方岩雄-肖业鹏\8.D
样品: 8

峰编号: 20 8.862 分钟处 面积: 7686124 面积 % 6.07

每个谱库中 3 个最匹配的记录。

	Ref\#	CAS\#	匹配度
D:\DATABASE\DEMO.L 未检索到匹配。			
C:\DATABASE\NIST11.L			
1 Cyclohexyl methylphosphonothiono...	58455	004241-34-3	37
2 1-Nonen-4-ol, 3,4-dimethyl-	38259	1000164-23-5	32
3 Fumaric acid, di(5-methoxy-3-met...	179734	1000330-65-7	32

谱库检索报告 - 化学工作站积分器

未知谱图基于顶点

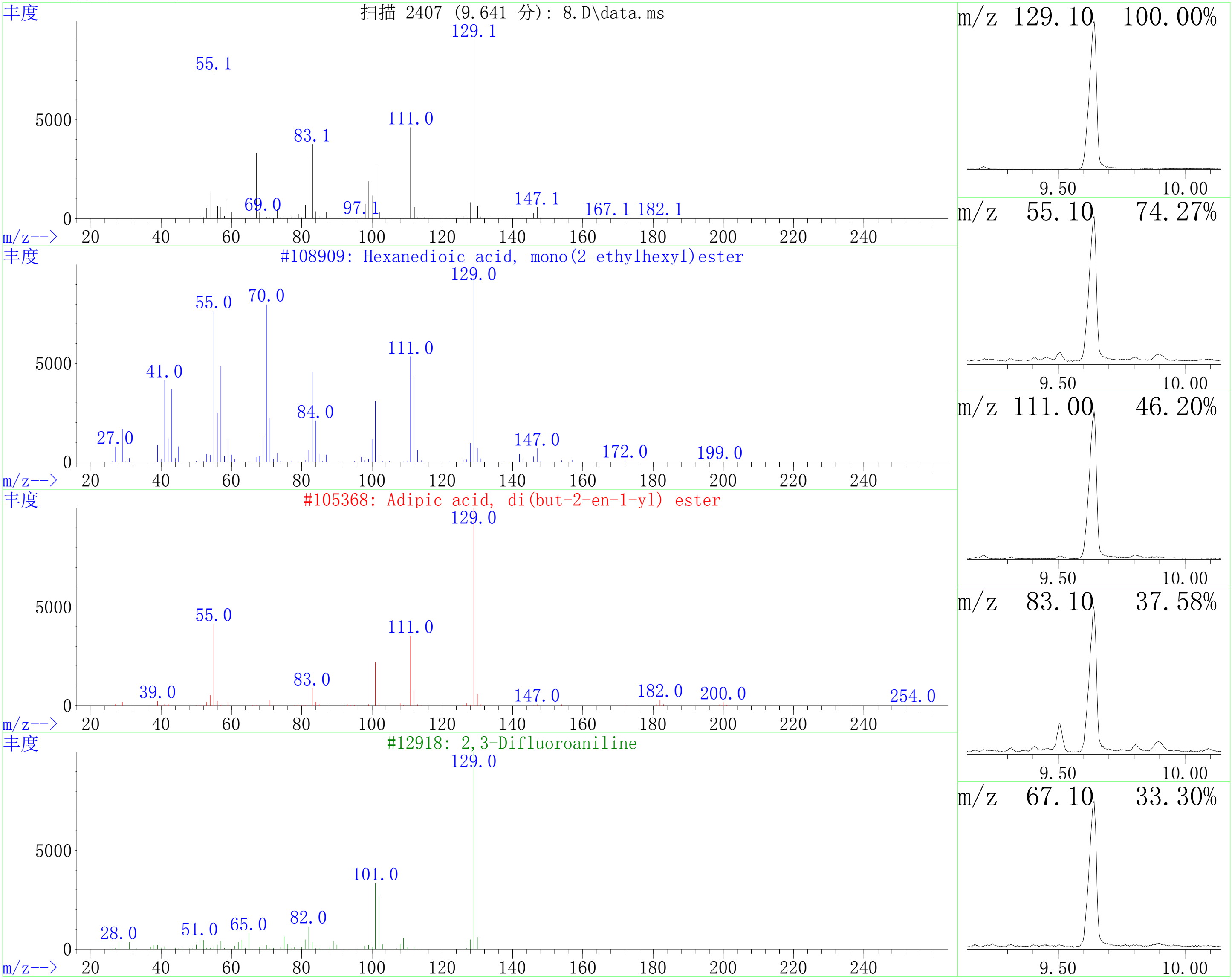

Data File: D:\msdchem\1\data\徐雄发\方岩雄-肖业鹏\8.D
样品: 8

峰编号: 21　9.641 分钟处　面积: 18738765　面积 % 14.80

每个谱库中 3 个最匹配的记录。	Ref\#	CAS\#	匹配度
D:\DATABASE\DEMO.L　未检索到匹配。			
C:\DATABASE\NIST11.L			
1 Hexanedioic acid, mono(2-ethylhe...	108909	004337-65-9	72
2 Adipic acid, di(but-2-en-1-yl) e...	105368	1000324-71-1	40
3 2,3-Difluoroaniline	12918	004519-40-8	38
